# Local glycolysis supports injury-induced axonal regeneration

**DOI:** 10.1083/jcb.202402133

**Published:** 2024-10-01

**Authors:** Luca Masin, Steven Bergmans, Annelies Van Dyck, Karl Farrow, Lies De Groef, Lieve Moons

**Affiliations:** 1 Department of Biology, Animal Physiology and Neurobiology Section, KU Leuven, Leuven Brain Institute, Leuven, Belgium; 2 Neuro-Electronics Research Flanders, Vlaams Instituut voor Biotechnologie, Leuven, Belgium; 3 imec, Leuven, Belgium

## Abstract

Successful axonal regeneration following injury requires the effective allocation of energy. How axons withstand the initial disruption in mitochondrial energy production caused by the injury and subsequently initiate regrowth is poorly understood. Transcriptomic data showed increased expression of glycolytic genes after optic nerve crush in retinal ganglion cells with the co-deletion of *Pten* and *Socs3*. Using retinal cultures in a multicompartment microfluidic device, we observed increased regrowth and enhanced mitochondrial trafficking in the axons of *Pten* and *Socs3* co-deleted neurons. While wild-type axons relied on mitochondrial metabolism, after injury, in the absence of *Pten* and *Socs3*, energy production was supported by local glycolysis. Specific inhibition of lactate production hindered injury survival and the initiation of regrowth while slowing down glycolysis upstream impaired regrowth initiation, axonal elongation, and energy production. Together, these observations reveal that glycolytic ATP, combined with sustained mitochondrial transport, is essential for injury-induced axonal regrowth, providing new insights into the metabolic underpinnings of axonal regeneration.

## Teaser

Axonal regeneration is an energy-demanding process. Masin et al. show that in *Pten* and *Socs3* co-deleted retinal ganglion cells, axonal regrowth requires and is fueled by a local axonal upregulation of glycolysis. Its targeting could provide new therapeutic avenues to induce axonal regrowth.

## Introduction

The adult mammalian central nervous system (CNS) has a very limited regenerative capacity. Upon injury, most neurons die and the few survivors fail to regrow their axons, which eventually degenerate leaving irreversible damage (Varadarajan et al., 2022; Fawcett, 2020). Regrowth is limited first by neuron-extrinsic mechanisms, such as glial scarring and the release of growth-inhibiting molecules following injury (Varadarajan et al., 2022; Fawcett, 2020; Williams et al., 2020). These include myelin debris, which binds to the axonal surface, blocking axonal regeneration. Second, even if these are cleared, the growth capacity of adult CNS neurons is extremely limited by neuron-intrinsic mechanisms, including the progressive decrease in expression of regeneration-associated genes during development (Williams et al., 2020; He and Jin, 2016; Mahar and Cavalli, 2018). Decades of research led to the discovery of genetic interventions that are able to partially counteract such inhibition (Varadarajan et al., 2022; Fawcett, 2020; Williams et al., 2020). One of these is the co-deletion of phosphatase and tensin homolog (*Pten*) and suppressor of cytokine signaling 3 (*Socs3*), which leads to robust axonal regrowth in both the optic nerve and spinal cord (Jin et al., 2015; Sun et al., 2011). Nonetheless, full circuit restoration and functional recovery remain unachievable.

Axonal regeneration is an extremely energy-demanding process, and over the years, mitochondria have emerged as critical players. The transport of functional mitochondria to the axons and their accumulation at the growth cone is crucial for regeneration (Cartoni et al., 2016, 2017; Zhou et al., 2016; Mar et al., 2014; Beckers et al., 2023). However, injury has been reported to depolarize the local population of mitochondria in axons (Zhou et al., 2016; Petrova et al., 2021), and sustained axonal ATP depletion is proposed to be a significant cause of Wallerian degeneration (Conforti et al., 2014; Hopkins et al., 2021). Unfortunately, mitochondria are predominantly stationary in the adult mammalian CNS, localizing at the site of synapses to provide the energy required for firing and releasing neurotransmitters (Lewis et al., 2016). As such, while remobilization of mitochondria is a viable strategy to sustain axonal regeneration, axons need to first withstand the injury, in the presence of a disrupted cytosolic environment and local dysfunctional mitochondria, before the allocation of healthy mitochondria to the growth cone takes place. Therefore, mitochondria-independent mechanisms might be at play after injury and might be required to initiate axonal regeneration.

Leveraging a previously published large single-cell RNA sequencing (scRNAseq) dataset of wild-type (WT) and genetically modified retinal ganglion cells (RGCs), in this study, we uncovered that RGCs co-deleted for *Pten* and *Socs3* promptly undergo a switch in the expression of metabolism-related genes after optic nerve crush injury. This includes the increased expression of antioxidant genes and, most importantly, the upregulation of glycolytic ones, which together with mitochondria could play a role in counteracting the energetic deficiency induced by axonal damage. Using an in vitro culture of postnatal retinal cells in microfluidic devices, we confirmed that injury-induced axonal regeneration of RGCs is associated with mitochondrial transport within the axons and that this is enhanced after injury upon deletion of *Pten* and *Socs3*. Interestingly, with functional assays using genetically encoded fluorescent biosensors, we found that *Pten*^*−*/*−*^;*Socs3*^*−/−*^ neurons upregulate glycolysis locally in the distal axonal compartment immediately after axotomy. Upstream downregulation of glycolysis via galactose treatment completely abolishes the enhanced regeneration phenotype of *Pten*^*−*/*−*^;*Socs3*^*−/−*^ axons and impairs their ability to produce ATP, while selective inhibition of lactate production impairs axonal survival and regrowth initiation only. This work demonstrates the importance of axonal glycolysis as a source of energy during not only injury survival but also in the initial phases of axonal regrowth.

## Results

### Optic nerve injury induces a transcriptomic shift toward glycolysis in *Pten* and *Socs3* co-deleted RGCs

**Figure 1.**
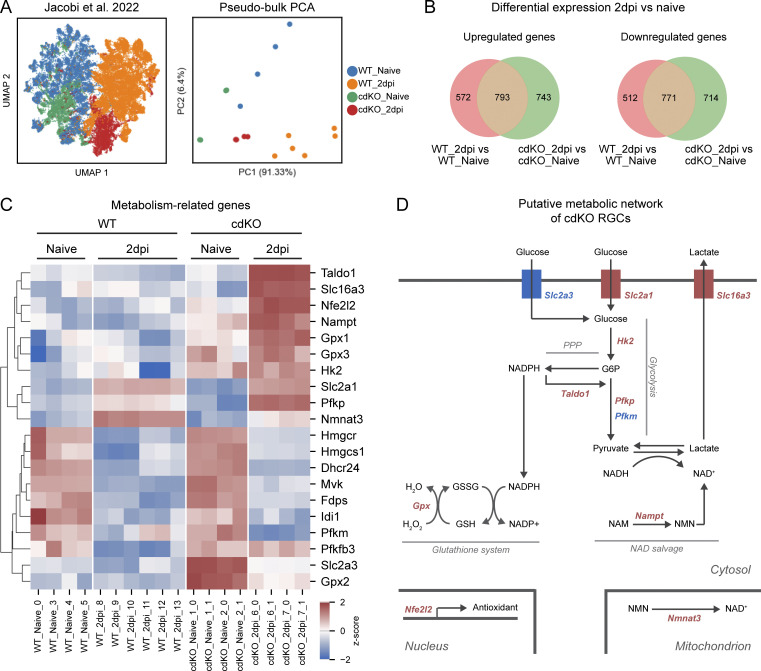
**Optic nerve injury induces a transcriptomic shift toward glycolysis in *Pten* and *Socs3* co-deleted RGCs. (A)** Pseudobulk expression matrices were generated from the Jacobi et al. scRNAseq dataset (Jacobi et al., 2022), selecting only WT RGCs and *Pten* and *Socs3* co-deleted RGCs with overexpression of CNTF (cdKO) in the uninjured condition (naive) and at 2 dpi time points. As expected, principal component analysis (PCA) shows the clustering of samples based on genotype and time point. **(B)** Differential gene expression on pseudobulk samples was performed with pyDESeq2 (Wald test) on injured RGCs at 2 dpi versus naive for both WT and cdKO RGCs. Genes were deemed differentially expressed if |log_2_FC| >1 and FDR <0.05. Venn diagrams representing the differentially expressed genes at 2 dpi show a partially overlapping but distinct response to injury between WT and cdKO RGCs. **(C)** Heatmap showing hierarchically clustered expression profiles of genes related to metabolism in WT and cdKO RGCs, both in the uninjured condition (naive) and at 2 dpi. Genes were selected among the ones differentially expressed at 2 dpi versus naive RGCs in both genotypes and the ones between cdKO and WT RGCs at 2 dpi. cdKO RGCs after optic nerve crush show an upregulated expression of genes involved in glycolysis (*Slc2a1*, *Hk2*, *Pfkp*, and *Slc16a3*) and antioxidant response (*Nfe2l2*, *Taldo1*, and *Gpx1-3*). Both WT and cdKO RGCs display downregulation of genes involved in the mevalonate pathway and cholesterol biosynthesis (*Hmgcr*, *Hmgcs1*, *Mvk*, *Fdps*, *Dhcr24*, and *Idi1*). **(D)** Putative metabolic network of injured cdKO RGCs inferred from the expression data. Upregulated genes are depicted in red, while downregulated in blue. Injury causes an increase in the flux of glycolysis and of the PPP. The latter generates NADPH, which is likely used for antioxidant responses. Finally, lactate generation and increased NAD salvage cooperate to restore NAD^+^ levels. Of note, the upregulation of *Slc2a1*, *Nmnat3*, and to a lesser extent *Pfkp* is shared with the WT genotype. GSH (glutathione, reduced form), GSSG (glutathione, disulfide form), NAM (nicotinamide).

To gain insights into the metabolic signature of regeneration-competent RGCs during injury response and initiation of axonal regrowth, we employed a previously published large-scale scRNAseq dataset by Jacobi et al. (2022). Their study aimed to evaluate the effect of three different genetic interventions, including the deletion of *Pten* and *Socs3*, on RGC survival and axonal regeneration. Here, we focused specifically on comparing WT RGCs with the ones subjected to the conditional double-knockout (cdKO) of *Pten* and *Socs3* at 2 days post-injury (dpi), the earliest time point available (Fig. 1 A). Of note, this double knockout was carried out together with the overexpression of *Cntf*, which is known to boost the effect of *Socs3* deletion (Smith et al., 2009; Sun et al., 2011; Xie et al., 2021). We performed the analysis on pseudobulk samples as this leads to more robust differential expression as compared with single-cell data (Squair et al., 2021; Murphy and Skene, 2022). This decision was also justified by the fact that, upon deletion of *Pten* and *Socs3*, most RGC subtypes were shown to regenerate (Jacobi et al., 2022), and as such we aimed to discover subtype-independent mechanisms rather than subtype-specific ones.

**Figure S1.**
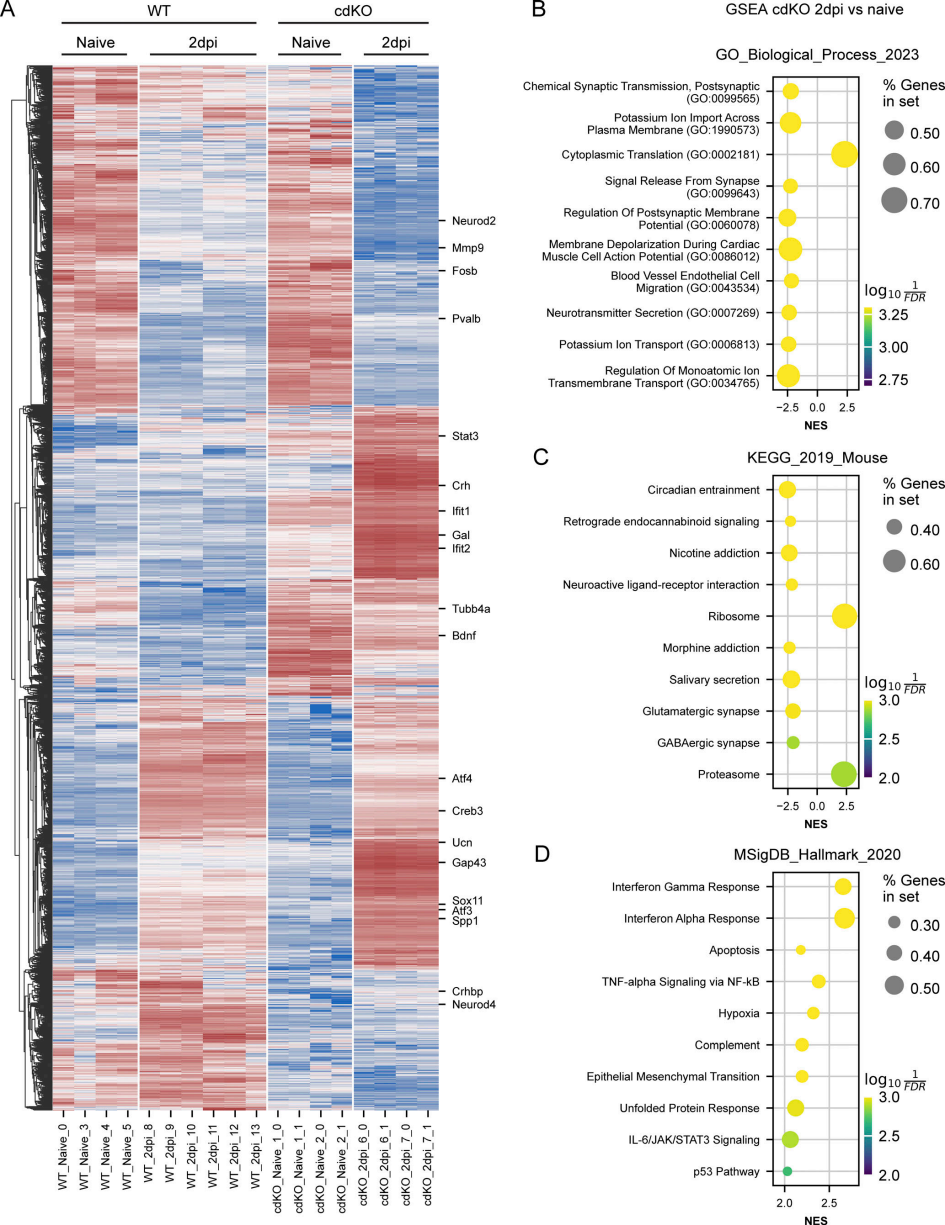
**Validation of the differential expression analysis. (A)** Hierarchically clustered heatmap of differentially expressed genes in WT and cdKO RGCs at 2 dpi versus uninjured ones (naive), from the Jacobi et al. (2022) dataset. Genes were deemed differentially expressed if |log_2_FC| >1 and FDR <0.05 (pyDESeq2, Wald test). The list was obtained by combining the outcome of the differential expression analysis between (1) cdKO RGCs at 2 dpi versus naive ones, (2) WT RGCs at 2 dpi versus naive ones, and (3) cdKO RGCs versus WT neurons at 2 dpi. Results recapitulate previous analysis by Jacobi et al. and previous literature. cdKO RGCs show specific upregulation of regeneration-associated genes such as *Gap43*, *Spp1*, *Stat3*, and *Gal* as compared to WT ones. On the other hand, cdKO RGCs display reduced expression as compared to WT of genes known to restrict regeneration, such as *Crhbp* and *Mmp9*. **(B–D)** Top 10 enriched pathways obtained from GSEA of cdKO RGCs at 2 dpi versus naive using the GO Biological Process, KEGG, and MSigDB Hallmark datasets. GSEA analysis was performed with GSEApy and pathways were considered significantly enriched with FDR < 0.05. In agreement with previous reports, cdKO RGCs at 2 dpi show enrichment of genes related to translation and ribosome, immune response, and STAT3 signaling. cdKO RGCs at 2 dpi also display enrichment of genes related to hypoxia, which is characterized by glycolytic metabolism. On the other hand, GSEA analysis shows negative enrichment of genes related to synaptic transmission at 2 dpi.

Differential expression analysis on pseudobulk samples with pyDESeq2 showed that WT and cdKO RGCs shared 793 upregulated genes at 2 dpi compared with naive, while 743 genes were exclusively upregulated in cdKO RGCs and 572 exclusively in WT ones (Fig. 1 B). On the other hand, WT and cdKO neurons shared 771 downregulated genes at 2 dpi compared with naive (Fig. 1 B). Additionally, 714 genes were selectively downregulated in cdKO RGCs and 512 only in WT RGCs (false discovery rate [FDR] < 0.05, |log2 fold change [FC]| > 1). Together this shows that injury causes a partially overlapping, but distinct transcriptional rearrangement in cdKO RGCs as compared with WT ones. Hierarchical clustering of the differential gene expression profiles and gene set enrichment analysis (GSEA) showed results reminiscent of previous analyses and comprising genes and pathways known to be involved in RGC survival and regeneration (Fig. S1, A–D) (Jacobi et al., 2022; Tran et al., 2019), validating the approach. Here, we focused on the outcome of the differential expression analysis between (1) cdKO RGCs at 2 dpi versus naive ones, (2) WT RGCs at 2 dpi versus naive ones, and (3) cdKO RGCs versus WT neurons at 2 dpi, with the goal to uncover gene expression signatures induced by injury in both phenotypes and those that are specific to the regeneration-competent cdKO condition. From the pool of differentially expressed genes, we selected the ones involved in energy production pathways as well as in related metabolic processes, such as antioxidant response and lipid metabolism (Fig. 1 C). Injury induced an upregulation of the glucose transporter *Slc2a1* (GLUT1) in both WT and cdKO RGCs. Additionally, only cdKO RGCs showed upregulated expression of the glycolytic genes *Pfkp* (PFK1, phosphofructokinase, platelet) and *Slc16a3* (MCT4, monocarboxylate transporter 4) at 2 dpi as compared with naive conditions (Fig. 1 C). PFK1 is the main rate-limiting enzyme of glycolysis, while MCT4 mediates the export of lactate derived from aerobic glycolysis (Warburg effect). Moreover, cdKO RGCs showed a constitutive upregulation of *Hk2* (HK2, hexokinase 2) as compared with WT ones, which was maintained after injury at 2 dpi, and a higher expression of *Pfkfb3* (PFKFB3/iPFK2, phosphofructokinase 2) at 2 dpi as compared with WT RGCs (Fig. 1 C). The expression of GLUT1, HK2, PFK1, and MCT4 has been reported to alter the maximal rate of glycolysis (Tanner et al., 2018). In the CNS, the expression of these genes is normally associated with glycolytic astrocytes as opposed to neurons, with the latter expressing instead the isoforms *Slc2a3* (GLUT3) and *Pfkm* (PFK1, muscle) under physiological conditions. The expression of these two genes was downregulated in cdKO RGCs after injury, but the expression of *Slc2a3* nonetheless remained higher in cdKO RGCs as compared with WT ones at 2 dpi (Fig. 1 C). Finally, we did not detect any substantial increase or decrease in the expression of genes involved in oxidative phosphorylation, with only a handful of the dozens of subunits of the mitochondrial electron transport chain showing differential expression at 2 dpi in both WT and cdKO RGCs (Table S1). Similarly, after injury, we did not identify any significant change in the expression of genes involved in mitochondrial dynamics, as in fusion/fission and trafficking (Table S1), suggesting that these are likely not controlled at the transcriptional level. These findings show that cdKO RGCs undergo a transcriptomic switch toward glycolysis after optic nerve crush injury and acquire a gene expression profile associated with Warburg-like aerobic glycolysis (Fig. 1 D).

Lactate production is a mechanism required to restore cytosolic NAD^+^ in the presence of impaired oxidative phosphorylation. Another related mechanism is the NAD salvage pathway, which was shown to be essential to prevent Wallerian degeneration after axonal damage in the peripheral nervous system (PNS) (Conforti et al., 2014; Hopkins et al., 2021) as well as in the optic nerve during glaucoma (Tribble et al., 2023; Petriti et al., 2021). At 2 dpi, we observed an upregulation of *Nmnat3* (nicotinamide nucleotide adenylyltransferase 3) in both WT and cdKO RGCs, and we detected an upregulation of *Nampt* (nicotinamide phosphoribosyltransferase) specifically in cdKO RGCs as compared to WT RGCs (Fig. 1 C). The latter constitutes the rate-limiting step in the conversion of nicotinamide into nicotinamide mononucleotide (NMN), while *Nmnat3* encodes for the mitochondrial isoform converting NMN into NAD^+^. Furthermore, we detected an upregulation of *Taldo1* (TALDO1, transaldolase 1) in cdKO RGCs at 2 dpi (Fig. 1 C). In cells not requiring nucleotide biosynthesis, this enzyme of the non-oxidative pentose phosphate pathway (PPP) controls the rate-limiting conversion of excess ribulose-6-phosphate into glycolytic intermediates after the generation of NADPH. One of the primary uses of NADPH within cells is for antioxidant response. Accordingly, we detected an upregulation of *Nfe2l2* (NRF2, nuclear factor erythroid 2-related factor 2), a master regulator of the antioxidant response, in cdKO RGCs at 2 dpi as compared with WT ones (Fig. 1 C). This was accompanied by a significantly higher expression of *Gpx2* (glutathione peroxidase 2, log2FC 1.88 versus WT) at 2 dpi in cdKO RGCs as compared with WT ones (Fig. 1 C), together with a minor increase in the expression of *Gpx1* and *Gpx3* (log2FC 0.92 and 0.68 versus WT, respectively). These are involved in the reduction of hydrogen peroxide into water, a reaction that is coupled with the reduction of glutathione, requiring NADPH. On the other hand, the NADPH-consuming mevalonate and cholesterol biosynthesis pathways showed the downregulation of many of their genes after injury in both WT and cdKO RGCs (Fig. 1 C). Inhibition of these pathways via statin treatment was previously reported to induce axonal regeneration in RGCs (Roselló-Busquets et al., 2019; Shabanzadeh et al., 2021). Collectively these data show that, after injury, as compared with WT ones, cdKO RGCs undergo a transcriptomic shift associated with metabolism. This involves the inferred switch toward a Warburg-like glycolytic phenotype, the upregulation of the NAD salvage pathway, and the strengthening of the antioxidant response machinery (Fig. 1 D).

### Lactate export is restricted to the early phases of regeneration in cdKO RGCs

**Figure 2.**
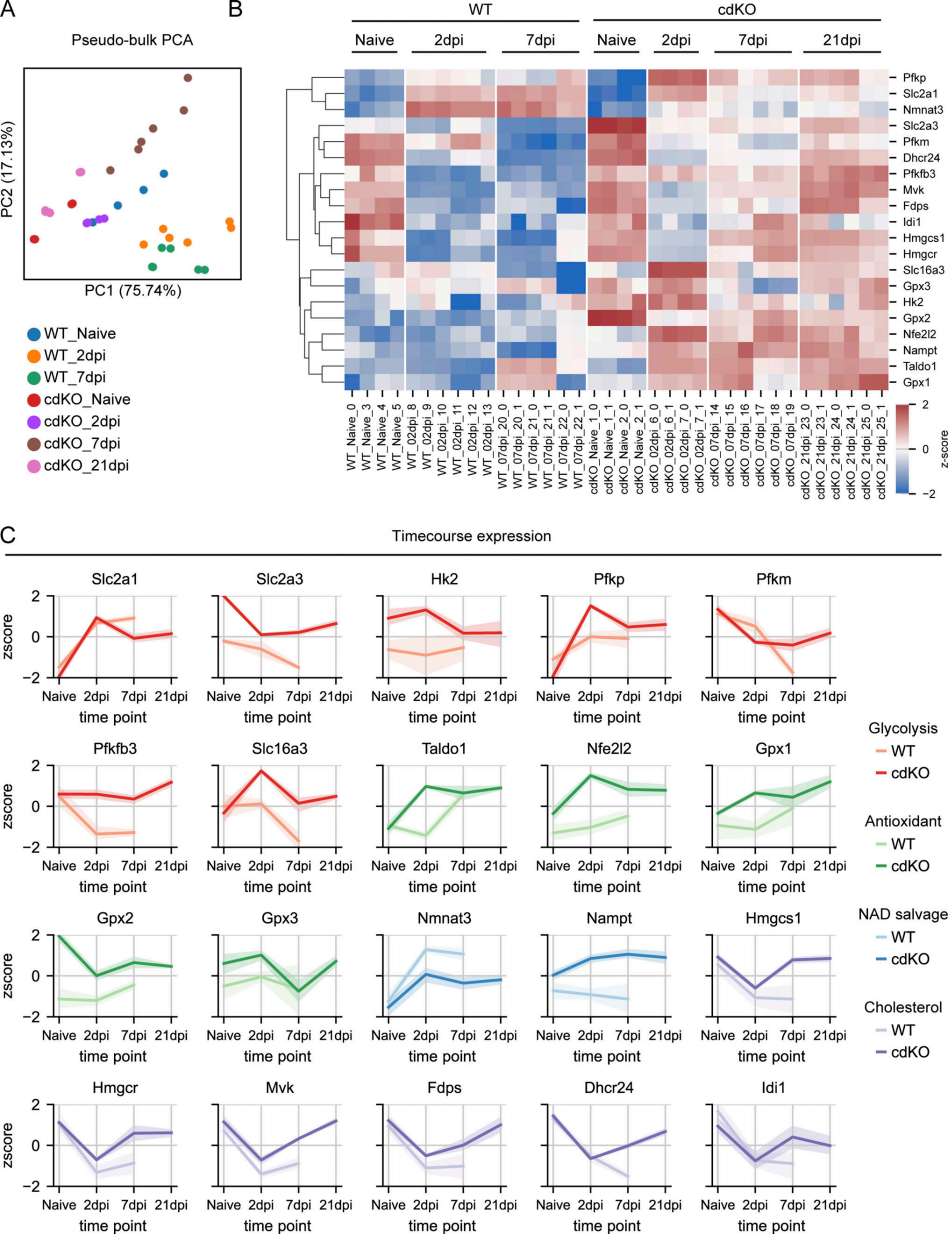
**Increased glycolysis is maintained during all phases of axonal regrowth, but lactate export is restricted to early phases only. (A)** Principal component analysis (PCA) of pseudobulk samples from the Jacobi et al. dataset (Jacobi et al., 2022), including both WT and cdKO RGCs at the naive, 2 and 7 dpi time points. Additionally, the 21 dpi was available only for the cdKO conditions and was included in the analysis. Principal component analysis shows regeneration-incompetent WT RGCs at 2 and 7 dpi segregating from their naive counterparts and from regeneration-competent cdKO RGCs. **(B)** Hierarchically clustered heatmap showing the expression profile at different time points after injury of genes related to metabolism in WT and cdKO RGCs. Genes were selected among the ones differentially expressed at 2 dpi versus naive RGCs in both genotypes and the ones between cdKO and WT RGCs at 2 dpi (pyDESeq2, |log_2_FC| >1 and FDR <0.05) (Fig. 1 C). **(C)** Relative expression (z-score) across time points in WT and cdKO RGCs of genes involved in glycolysis and Warburg-related lactate export (red), NADPH generation and antioxidant response (green), NAD salvage (blue), and cholesterol biosynthesis (purple) that were identified as differentially expressed at the 2 dpi time point. In cdKO RGCs, the expression of glycolytic, antioxidant and NAD salvage genes is increased at 2 dpi as compared to naive and maintained until 21 dpi, while the expression of the lactate transporter Slc16a3 is increased only at 2 dpi. On the other hand, WT RGCs do not show this generalized trend, with only *Slc2a1* and *Nmnat3* showing significantly upregulated expression at 2 and 7 dpi. The expression of genes involved in cholesterol biosynthesis is downregulated at 2 dpi in both genotypes but is recovered at 21 dpi in cdKO RGCs.

We next assessed whether the transcriptomic shift detected at 2 dpi would be conserved in later phases of regeneration during axonal elongation in the optic nerve and chiasm. For this, we generated pseudobulk matrices of WT and cdKO RGCs at the naive, 2 and 7 dpi time points. Additionally, the Jacobi dataset included the 21 dpi time point for cdKO RGCs (Fig. 2 A). The expression of *Slc2a1* and the rate-limiting *Pfkp* peaked at 2 dpi but remained significantly upregulated until 21 dpi in cdKO RGCs (Fig. 2, B and C). Conversely, the expression of *Slc16a3* was significantly upregulated only at 2 dpi (Fig. 2, B and C), suggesting that increased glycolysis is a feature of regenerating RGCs, but that lactate export might primarily be a characteristic of early injury response and regrowth initiation only. Compared with WT RGCs, cdKO ones showed a sustained trend of higher expression of glycolytic genes, with *Slc2a3*, *Pfkfb3*, and *Slc16a3* being differentially expressed at 7 dpi in cdKO versus WT RGCs (Fig. 2, B and C). Similar to glycolysis, the expression of *Taldo1* and the antioxidant transcription factor *Nfe2l2* was increased from 2 dpi until 21 dpi in cdKO RGCs as compared with uninjured RGCs (Fig. 2, B and C). Regarding the NAD salvage genes, *Nampt* and *Nmnat3* showed a sustained trend of upregulated expression until 21 dpi in cdKO RGCs, with *Nampt* being differentially expressed at 7 dpi in cdKO versus WT RGCs. A different pattern of expression was detected for the genes related to the mevalonate and cholesterol biosynthesis pathway. While their expression was significantly downregulated at 2 dpi, it was recovered at 21 dpi in cdKO RGCs (Fig. 2, B and C), suggesting that they might be required for later phases of regeneration. Collectively, this data indicates that increased glucose metabolism and glycolysis are necessary for all phases of regeneration, from injury response and axonal survival to regrowth initiation and elongation. Lactate generation and export on the other hand could be a feature only of the early phases and as such it could constitute a mechanism of axonal survival.

### A microfluidic platform for the study of axonal bioenergetics during injury-induced regrowth

**Figure S2.**
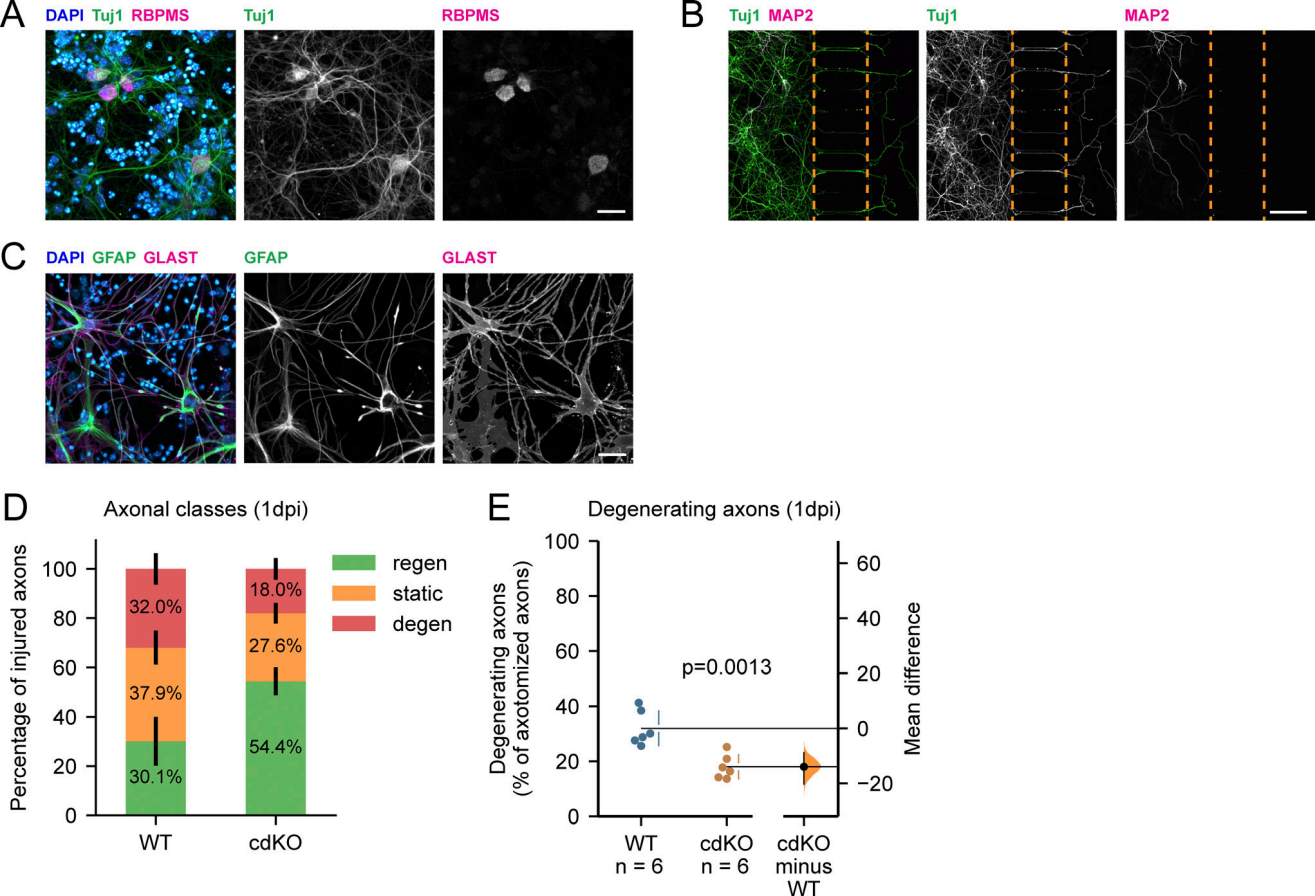
**Validation of the retinal cell culture and microfluidic model. (A)** Immunocytochemistry for DAPI, Tuj1, and RBPMS at 14DIV identifies RGCs, co-labeled by both markers, in the mixed retinal cell culture. Scale bar 25 µm. **(B)** Immunocytochemistry for Tuj1 and MAP2 at 14DIV identifies Tuj1^+^ RGCs in the somatic compartment and their axons in the axonal one. Importantly, the dendritic marker Map2 is restricted to the somatic compartment. The edges of the microgrooves are delineated by the orange dotted line. Scale bar 100 µm. **(C)** Immunocytochemistry for DAPI, GFAP, and GLAST at 14DIV reveals the presence of retinal macroglia, co-labeled by both markers. Scale bar 25 µm. **(D)** Graphical representation of the percentages of regenerating (green, bottom), static (orange, middle), and degenerating (red, top) axons for WT and cdKO neurons at 1 dpi. Classification is performed as follows: (1) regenerating axons extend past the site of injury, (2) degenerating axons either fully degenerate or show substantial beading and rupture, and (3) static axons show minimal or no sign of beading but fail to extend past the site of injury. **(E)** Quantification of degenerating axons in the axonal compartment at 1 dpi reveals a significant decrease in the percentage of degenerating axons in cdKO neurons. Data from six (D and E) independent experiments, presented as mean ± SD (D and E) and bootstrap 95% confidence interval versus WT (E). Student’s *t* test (E). P values are reported within the graphs.

Large-scale metabolic changes in cells are orchestrated at the transcriptional level by altering the expression of rate-limiting enzymes. However, this cannot account for the local metabolic needs in different neuronal compartments or for changes at rapid timescales. Local metabolic control occurs posttranslationally through the subcellular localization and allosteric control of glycolytic enzymes (Wegner et al., 2015) and via dynamic mitochondrial positioning along axons, mediated by proteins like syntaphilin and Miro (Huang et al., 2021; Lin and Sheng, 2015). Studying these processes requires longitudinal assessment of the neurons, live imaging, and precise metabolic interventions (Koveal et al., 2020; Liu et al., 2017). All of these are particularly challenging in vivo within the optic nerve. Therefore, to study the metabolism driving axonal regeneration, we have developed an in vitro microfluidic system using primary retinal cell cultures from postnatal *Pten*^*fl/fl*^;*Socs3*^*fl/fl*^ mice. Multicompartment microfluidics enable the separation of neuronal cell bodies in the somatic channel from their axons, which extend through microgrooves into the axonal channel (Taylor et al., 2005). This fluidic isolation enables clean axonal injuries and independent treatment of the axonal and somatodendritic compartments. 2 wk into culture, we identified RGCs in the somatic channel via Tuj1 and RBPMS (RNA Binding Protein, MRNA Processing Factor) labeling (Fig. S2 A), both markers of RGCs in mice (Rodriguez et al., 2014; Nadal-Nicolás et al., 2023). Dendrites were confined to the somatic channel, while axons extended into the axonal channel, as revealed by MAP2 and Tuj1 labeling (Fig. S2 B). Finally, retinal astrocytes and Müller glia were also detected in the somatic channel using GFAP (Glial Fibrillary Acidic Protein) and GLAST (Glutamate/apartate Transporter) labeling (Fig. S2 C).

**Figure 3.**
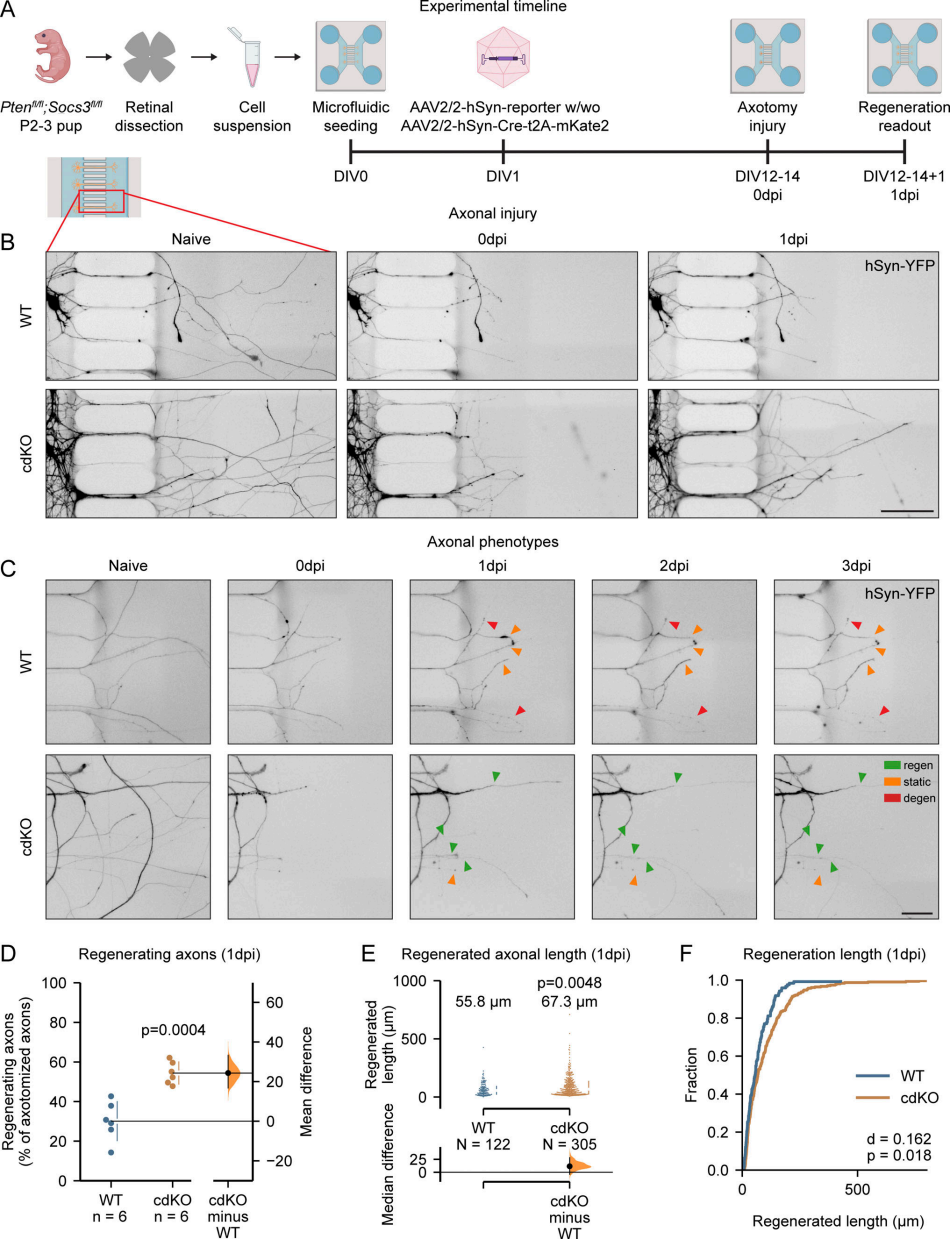
**Deletion of *Pten* and *Socs3* increases axotomy-induced axonal regeneration in primary RGCs. (A)** Primary retinal cultures are obtained from retinae of *Pten*^*fl/fl*^*;Socs3*^*fl/fl*^ mice at P2–3 and seeded in microfluidic devices. The RGCs are labeled and conditionally deleted for *Pten* and *Socs3* via AAV2/2-mediated transduction at 1DIV. Axonal injury is performed between 12DIV and 14DIV and the outcome of regeneration is assessed 1 day later (1 dpi, DIV12–14+1). **(B)** Representative images of uninjured axons (naive), injured axons immediately after axotomy (0 dpi), and regrowing axons (1 dpi) of WT and cdKO RGCs. hSyn-YFP is encoded by an AAV2/2-hSyn-ATeam^YEMK^-WPRE-hGHp vector (see Materials and methods). Scale bar 100 µm. **(C)** Representative images of degenerating axons (red), regenerating axons (green), and static axons (orange). The latter survive injury but do not grow past the cut site at 1 dpi and until 3 dpi. Scale bar 50 µm. **(D)** Quantification of the axons regrowing in the axonal compartment shows that deletion of *Pten* and *Socs3* induces an increase in the percentage of regenerating axons at 1 dpi. **(E)** Quantification of the axonal length shows that codeletion of *Pten* and *Socs3* induces a significant increase in the average length of regrowing axons at 1 dpi. The median length is reported above both groups. **(F)** Cumulative distribution of axonal lengths shows that *Pten* and *Socs3* co-deletion increases the length of regrowth at 1 dpi. Data from six independent experiments, presented as mean ± SD (D) or median ± 25–75th confidence interval (E) and bootstrap 95% confidence interval versus WT. Student’s *t* test (D), Kolmogorov–Smirnov test (E), and Mann–Whitney U test (F). P values are reported within the graphs.

**Figure S3.**
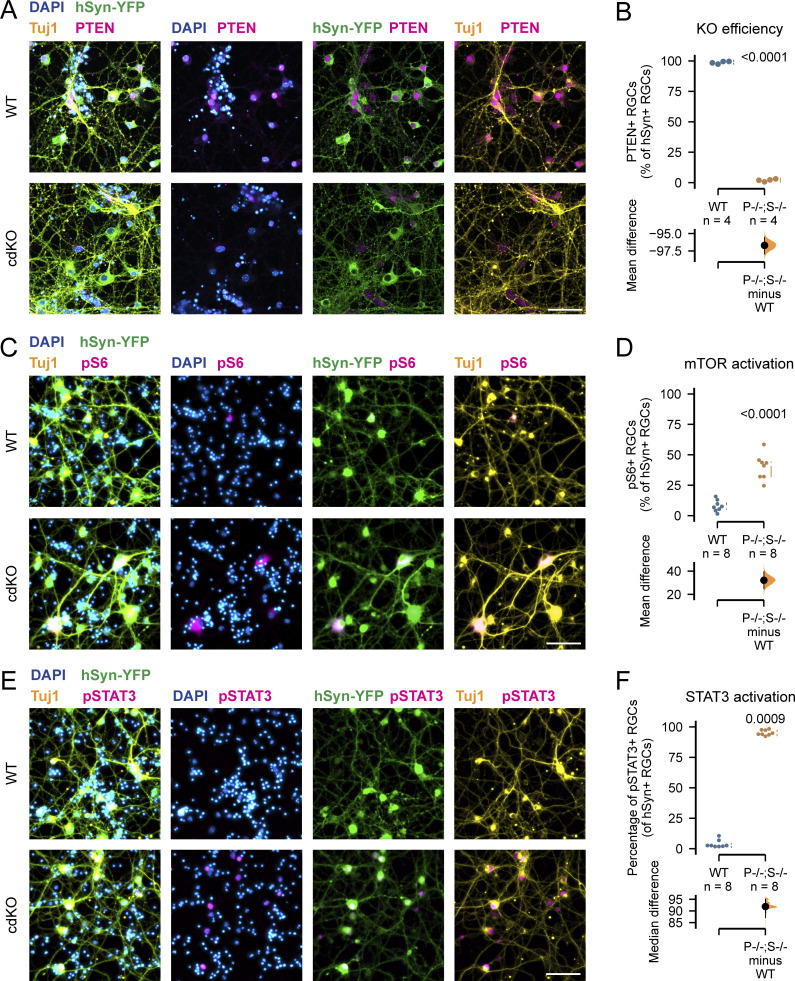
**AAV-mediated conditional knockout of *Pten* and *Socs3* leads to specific deletion and upregulation of regeneration-associated pathways at 14DIV. (A)** Immunolabelling for DAPI, PTEN, and Tuj1 shows specific loss of PTEN within Tuj1^+^/hSyn-YFP^+^ RGCs upon transduction with an AAV2/2-Syn-Cre viral vector (cdKO) as compared to WT cells, indicating specific recombination and gene knockout. Scale bar 50 µm. **(B)** Quantification of PTEN signal in the somata of hSyn-YFP^+^ RGCs shows that on average 98.7% of the RGCs are positive for PTEN at 14DIV in the WT condition as compared to only 2% in the cdKO culture, indicating that an efficient knockout is achieved. **(C)** Immunolabeling for DAPI, pS6, and Tuj1 reveals increased phosphorylation of S6 within Tuj1^+^/hSyn-YFP^+^ RGCs upon transduction with an AAV2/2-Syn-Cre viral vector as compared with WT cells, indicating mTOR activation. Scale bar 50 µm. **(D)** Quantification of pS6 signal in the somata of hSyn-YFP^+^ RGCs at 14DIV shows a ∼30% increase in the number of pS6^+^ RGCs upon deletion of *Pten* (P) and *Socs3* (S). **(E)** Immunolabeling for DAPI, pSTAT3, and Tuj1 reveals increased phosphorylation of STAT3 within Tuj1^+^/hSyn-YFP^+^ RGCs upon transduction with an AAV2/2-Syn-Cre viral vector as compared to WT cells, indicating JAK/STAT activation. Scale bar 50 µm. **(F)** Quantification of pSTAT3 signal in the somata of hSyn-YFP^+^ RGCs at 14DIV shows a ∼90% increase in the number of pSTAT3^+^ RGCs upon deletion of *Pten* and *Socs3*. In all experiments hSyn-YFP was encoded by *AAV2/2-hSyn1-ATeam*^*YEMK*^*-WPRE-hGHp*; see Materials and methods. Data from four (A and B) or two (C–F) independent experiments, presented as mean ± SD (B, D, and F) and bootstrap 95% confidence interval versus WT (B, D, and F). Student’s *t* test (B, D, and F). P values are reported within the graphs.

To elucidate the mechanisms underlying the regeneration induced by *Pten* and *Socs3* deletion, we divided the retinal cultures into two conditions: *Pten*^*fl/fl*^;*Socs3*^*fl/fl*^ cultures with no induced recombination (WT) and cultures in which we induced the cdKO in RGCs via AAV-mediated (Adeno-associated virus) Cre recombination (using an *AAV2/2-hSyn1-Cre-t2A-mKate2* viral vector) (Fig. 3 A). At 14 days in vitro, PTEN (Phosphatase And Tensin Homolog) immunolabeling was not detectable in 98% of the RGC somata, which were co-labeled by hSyn-YFP and Tuj1 (Fig. S3, A and B), highlighting efficient recombination with a high degree of specificity for RGCs. As expected, the deletion of *Pten* and *Socs3* in cdKO RGCs led to the activation of the regeneration-associated pathways mTOR and JAK/STAT3, assessed via labeling of pS6 and pSTAT3, respectively (Fig. S3, C–F). Notably, the degree of activation of mTOR and STAT3 measured in WT RGCs at 14 days in vitro (DIV) matched very closely the observations reported in vivo in adult animals (Leibinger et al., 2013a, 2013b; Park et al., 2008; Duan et al., 2015) (Fig. S3, C–F). As such, despite being harvested from mouse pups, the cultured RGCs closely resembled adult ones in terms of regeneration-associated signaling.

### Deletion of *Pten* and *Socs3* increases axonal regeneration of RGCs in vitro

To quantify the regeneration capacity of WT and cdKO RGCs, we performed an axotomy in the axonal channel and classified the axons in three groups at 1 dpi (Fig. 3, B and C). The three classes were (1) regenerating axons, which extended past the site of injury, (2) degenerating axons, which either fully degenerated or showed substantial beading and rupture, and (3) static axons, which showed minimal or no sign of beading, but failed to extend past the site of injury at 1 dpi. Of note, when followed up until 3 dpi, axons classified as static on 1 dpi failed to show either signs of degeneration or growth past the site of injury (Fig. 3 C). The relative percentages of these axon classes varied upon codeletion of *Pten* and *Socs3* (Fig. S2 D), with a significant increase in the percentage of regenerating axons at 1 dpi for cdKO RGCs as compared with WT (Fig. 3 D). Besides the increased regeneration, the deletion of *Pten* and *Socs3* led to a decrease in the percentage of degenerating axons at 1 dpi as compared with WT (Fig. S2 E). Finally, we also detected an increase in the median length of the regrown axon tracts at 1 dpi in cdKO RGCs as compared with WT neurons (Fig. 3, E and F). Overall, this demonstrates that the microfluidic setup allows the study of all major phases of axonal regeneration, namely injury survival and regrowth initiation, as well as early elongation. Moreover, these findings show that deletion of *Pten* and *Socs3* enhances both axonal survival and regeneration in cultured RGCs.

### Axonal regeneration is associated with mitochondrial transport and integrity

**Figure 4.**
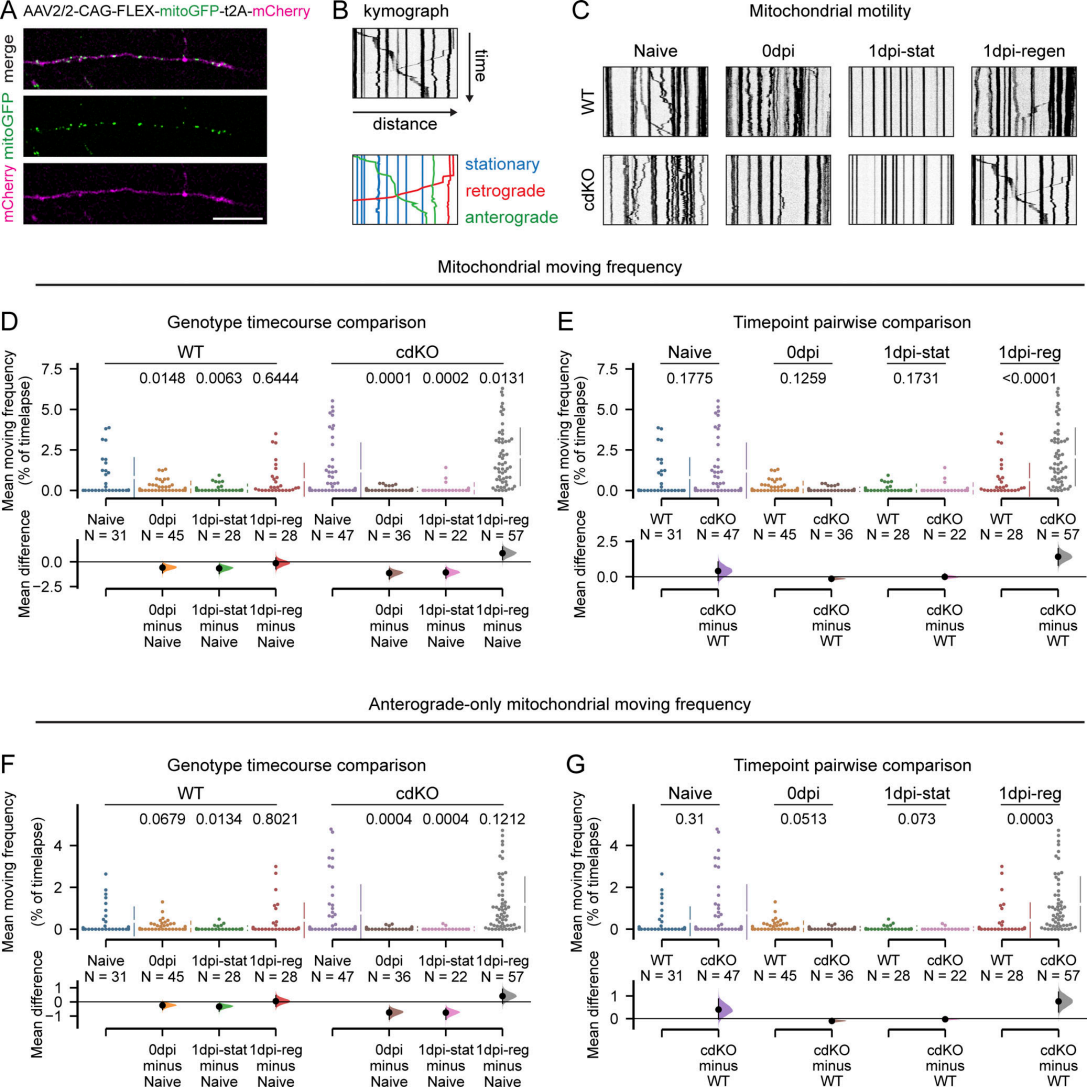
**Axonal regeneration is associated with the restoration of mitochondrial transport. (A)** RGC axons and their mitochondria are labeled via viral expression of mCherry and a mitochondrially targeted GFP, respectively. Scale bar 25 µm. **(B)** Example kymograph highlighting mitochondria that are classified as stationary (blue), transported retrogradely (red), and anterogradely (green). **(C)** Representative kymographs showing mitochondrial transport in the uninjured condition (naive), immediately after axotomy (0 dpi), and at 1 dpi, for both WT and cdKO axons. **(D)** Quantification of mitochondrial moving frequency per axon reveals that transport is reduced immediately after injury (0 dpi) in both genotypes. At 1 dpi, only regenerating axons show a similar (WT) or significantly higher (cdKO) mitochondrial transport as compared to naive ones. **(E)** Pairwise comparison of mitochondrial moving frequency between WT and cdKO axons at each time point and condition shows a comparable moving frequency between naive WT and cdKO axons, as well as at 0 and 1 dpi in static axons. On the other hand, regenerating cdKO axons at 1 dpi reveal a significantly higher mitochondrial moving frequency as compared with their WT counterparts. **(F)** Quantification of anterograde-only mitochondrial moving frequency per axon shows a pattern comparable with bidirectional movement. Regenerating axons show a similar (WT) or significantly higher (cdKO) mitochondrial transport as compared with naive ones. **(G)** Pairwise comparison of anterograde-only mitochondrial moving frequency between WT and cdKO axons at each time point and condition. Regenerating cdKO axons at 1 dpi reveal a significantly higher mitochondrial moving frequency as compared to their WT counterparts. Data from four independent experiments, presented as mean ± SD (D–G) and bootstrap 95% confidence interval versus relative naive (D and F) or versus WT (E and G). Welch ANOVA (D and F) or Mann–Whitney U test (E and G). P values are reported within the graphs.

Mitochondrial transport into the axons has been shown to be important for regeneration in other models (Zhou et al., 2016), and the deletion of *Pten* and *Socs3* was reported to be associated with increased axonal transport of mitochondria in embryonic cortical neurons (Cartoni et al., 2017). To determine whether this is also true in our RGC setup, we transduced them with a vector encoding a mitochondrially targeted GFP to label the mitochondria, as well as a cytosolic mCherry to visualize the whole neuron (*AAV2/2-CAG-FLEx-mitoGFP-t2A-mCherry-WPRE*) (Fig. 4 A). The usage of a flip-excision mechanism (FLEx) (Schnütgen et al., 2003) conferred Cre-dependent conditional expression and thus ensured that the expression of the reporter construct would occur only in cells that underwent Cre-dependent genetic deletion in a *Pten*^*fl/fl*^*;Socs3*^*fl/fl*^ background. We quantified the transport of mitochondria in the distal end of the axon via kymographic analysis (Fig. 4 B). This was performed in uninjured axons (naive) within the first hour after injury (0 dpi) and 1 dpi. Axonal injury caused a disruption of mitochondrial transport in both WT and cdKO axons, and trafficking was not restored in static axons at 1 dpi (Fig. 4, C and D). Regenerating WT axons at 1 dpi displayed transport comparable with their uninjured counterparts, while cdKO regenerating axons showed upregulated trafficking compared with naive (Fig. 4 D) and regenerating WT axons (Fig. 4 E). A similar pattern and genotypic difference in regenerating axons was detected when measuring anterograde transport only (Fig. 4, F and G).

**Figure 5.**
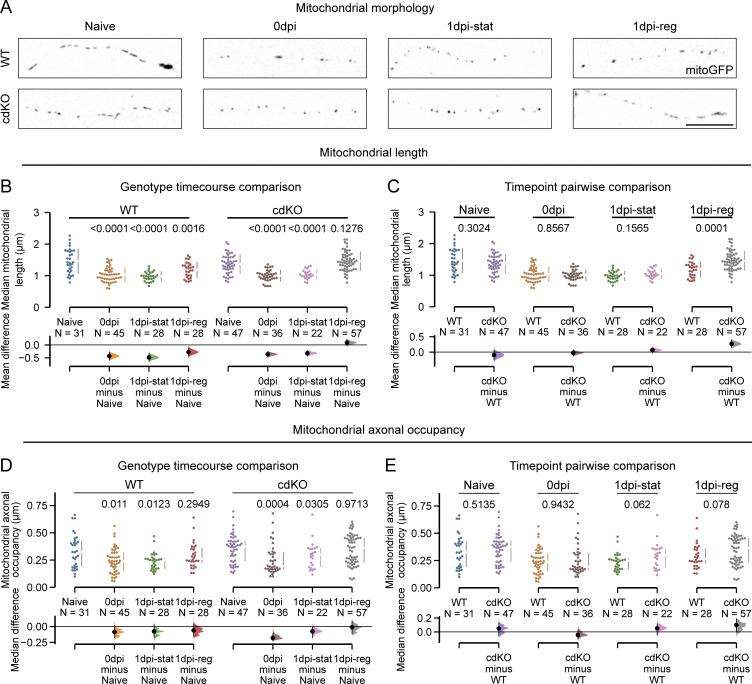
**Axonal regeneration is associated with the restoration of mitochondrial morphology and mass. (A)** Representative images of mitochondrial morphology in WT and cdKO axons, showing small round mitochondria immediately after injury at 0 dpi and in static axons at 1 dpi. Unlike cdKO axons, which at 1 dpi present an elongation of mitochondria comparable to uninjured ones, WT regenerating ones do not show a complete restoration of mitochondrial morphology at this time point. Scale bar 20 µm. **(B)** Quantification of the average mitochondrial length per axon reveals significantly shorter mitochondria in both genotypes at 0 dpi and in static axons. At 1 dpi, only regenerating cdKO axons restore mitochondrial lengths comparable with naive ones. **(C)** Pairwise comparison of mitochondrial length between WT and cdKO axons at each time point and condition discloses a comparable size of mitochondria between naive WT and cdKO axons, as well as at 0 and 1 dpi in static axons. On the contrary, regenerating cdKO axons at 1 dpi show a significantly higher mitochondrial length as compared to WT ones. **(D)** Quantification of mitochondrial axonal occupancy shows the recovery of mitochondrial mass in regenerating axons, but not in static ones, for both WT and cdKO RGCs. **(E)** Pairwise comparison of mitochondrial occupancy between WT and cdKO axons at each time point and condition shows no significant difference in axonal mitochondrial mass between WT and cdKO axons in any condition or time point. Data from four independent experiments, presented as mean ± SD (B and C) or median ± 25–75th confidence interval (D and E) and bootstrap 95% confidence interval versus relative naive (B and D) or versus WT (C and E). Welch ANOVA (B), Kruskal–Wallis ANOVA (D), or Mann–Whitney U test (C and E). P values are reported within the graphs.

Furthermore, axonal injury caused a significant decrease in mitochondrial length in both WT and cdKO axons at 0 dpi, leading to small, round mitochondria, possibly due to injury-induced fission (Fig. 5, A and B). At 1 dpi, only cdKO regenerating axons were able to fully recover mitochondrial length (Fig. 5 B), which was significantly higher than in regenerating WT axons (Fig. 5 C). On the other hand, static axons of both WT and cdKO RGCs did not restore an elongated mitochondrial morphology comparable with naive (Fig. 5 B). Lastly, as a measure of mitochondrial mass, we calculated the mitochondrial axonal occupancy (Lewis et al., 2018). As for mitochondrial length, we measured a small but significant decrease in occupancy after injury at 0 dpi, which was not restored at 1 dpi in static axons of both WT and cdKO RGCs (Fig. 5 D). Regenerating axons, on the other hand, restored occupancy to a value comparable with their naive counterparts in both genotypes (Fig. 5 D). Notably, no significant difference in mitochondrial transport (Fig. 4, E and G), morphology, and occupancy (Fig. 5, C and E) was measured in uninjured axons between WT and cdKO RGCs, suggesting that enhanced mitochondrial dynamics, unlike for embryonic cortical neurons (Cartoni et al., 2017), are not an intrinsic feature of relatively more matured cdKO RGCs. On the other hand, collectively, these data show that axonal regeneration is associated with mitochondrial transport and integrity, and the latter are enhanced after injury by deletion of *Pten* and *Socs3* in RGCs.

### Preservation of energy levels in cdKO axons is supported by glycolysis

**Figure 6.**
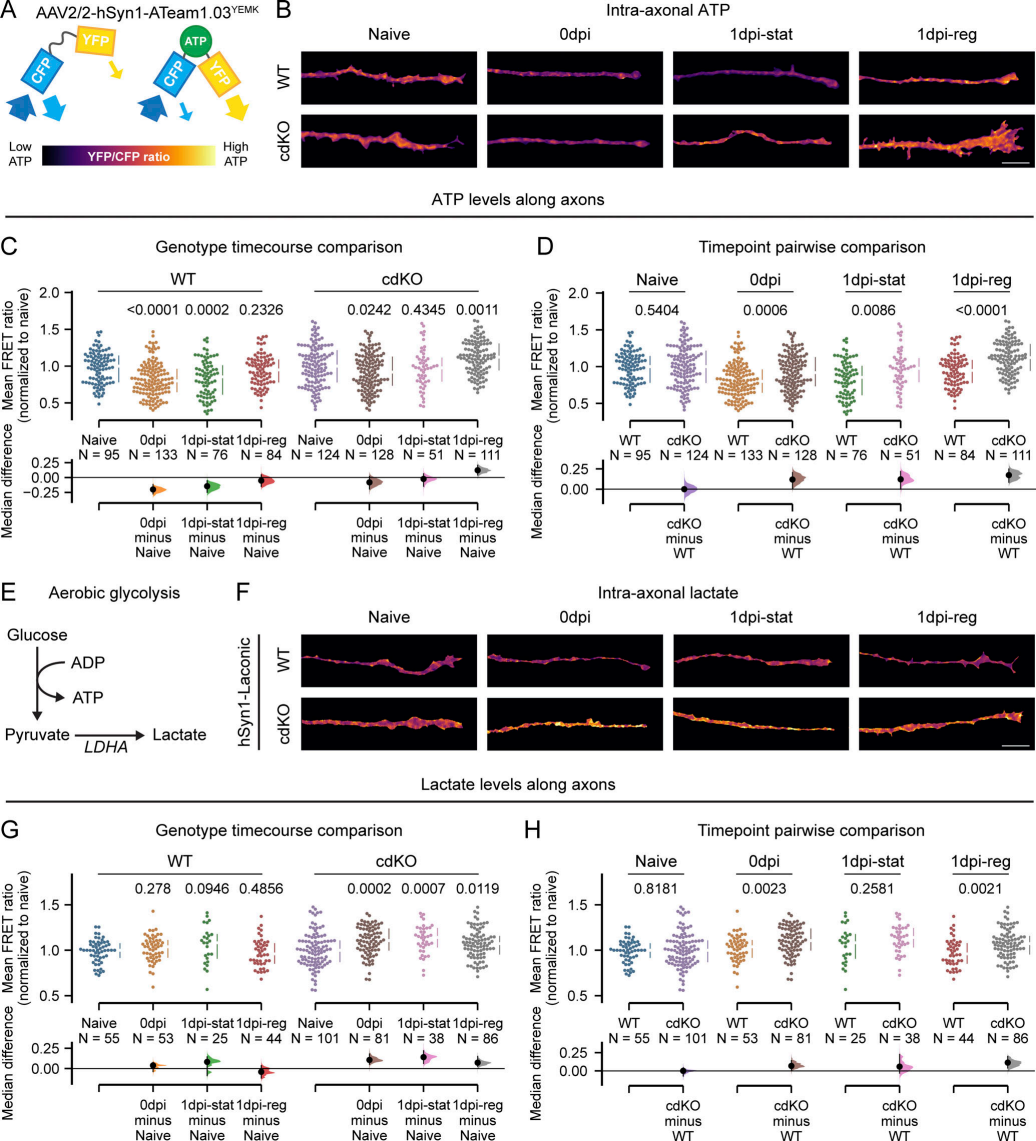
**Regeneration of cdKO axons is accompanied by increased ATP concentration, supported by glycolysis. (A)** Graphical representation of the ATeam biosensor. The binding of ATP causes a conformation shift, which allows FRET from CFP to YFP, in turn increasing the YFP/CFP FRET emission ratio (Imamura et al., 2009). The latter is proportional to the intra-axonal concentration of ATP. **(B)** Representative images of axonal endings of WT and cdKO neurons, depicted as pseudocolor representations of the FRET ratio of the ATP biosensor ATeam1.03^YEMK^. Scale bar 10 µm. **(C)** Quantification of the intra-axonal ATP concentration along the axon shows restoration of ATP only in regenerating axons for WT neurons and in both static and regenerating axons for cdKO neurons. **(D)** Pairwise comparison of intra-axonal ATP concentration between WT and cdKO axons at each time point reveals a significantly higher concentration of ATP in cdKO axons as compared with WT ones right after injury at 0 dpi and at 1 dpi, in both static and regenerating axons. Data points were normalized against the median of their relative naive uninjured control per independent experiment. The comparison of the non-normalized naive groups is reported in Fig. S4 A. **(E)** Schematic representation of aerobic glycolysis (Warburg effect), resulting in reduction of pyruvate into lactate via LDHA activity rather than import into mitochondria. **(F)** Representative images of axonal endings of WT and cdKO neurons, depicted as pseudocolor representations of the FRET ratio of the ATP biosensor Laconic. Here, the binding of lactate to the biosensor leads to a decrease in FRET efficiency, in turn increasing the CFP/YFP ratio. Scale bar 10 µm. **(G)** Quantification of axonal intracellular lactate concentration reveals upregulated aerobic glycolysis in cdKO axons following injury and at 1 dpi, regardless of regeneration outcome. This is not the case in WT RGC axons. **(H)** Pairwise comparison of intra-axonal lactate concentration between WT and cdKO axons at each time point discloses a significantly higher concentration of lactate in cdKO axons as compared with WT right after axotomy at 0 dpi and at 1 dpi in regenerating axons. Datapoints were normalized against the median of their relative naive uninjured control per independent experiment. The comparison of the non-normalized naive groups is reported in Fig. S4 A. Data from five (C and D) or four (G and H) independent experiments, presented as median ± 25–75th confidence interval and bootstrap 95% confidence interval versus relative naive (C and G) or versus WT (D and H). Kruskal-Wallis ANOVA (C and G), Mann–Whitney U test (D and H). P values are reported within the graphs.

**Figure S4.**
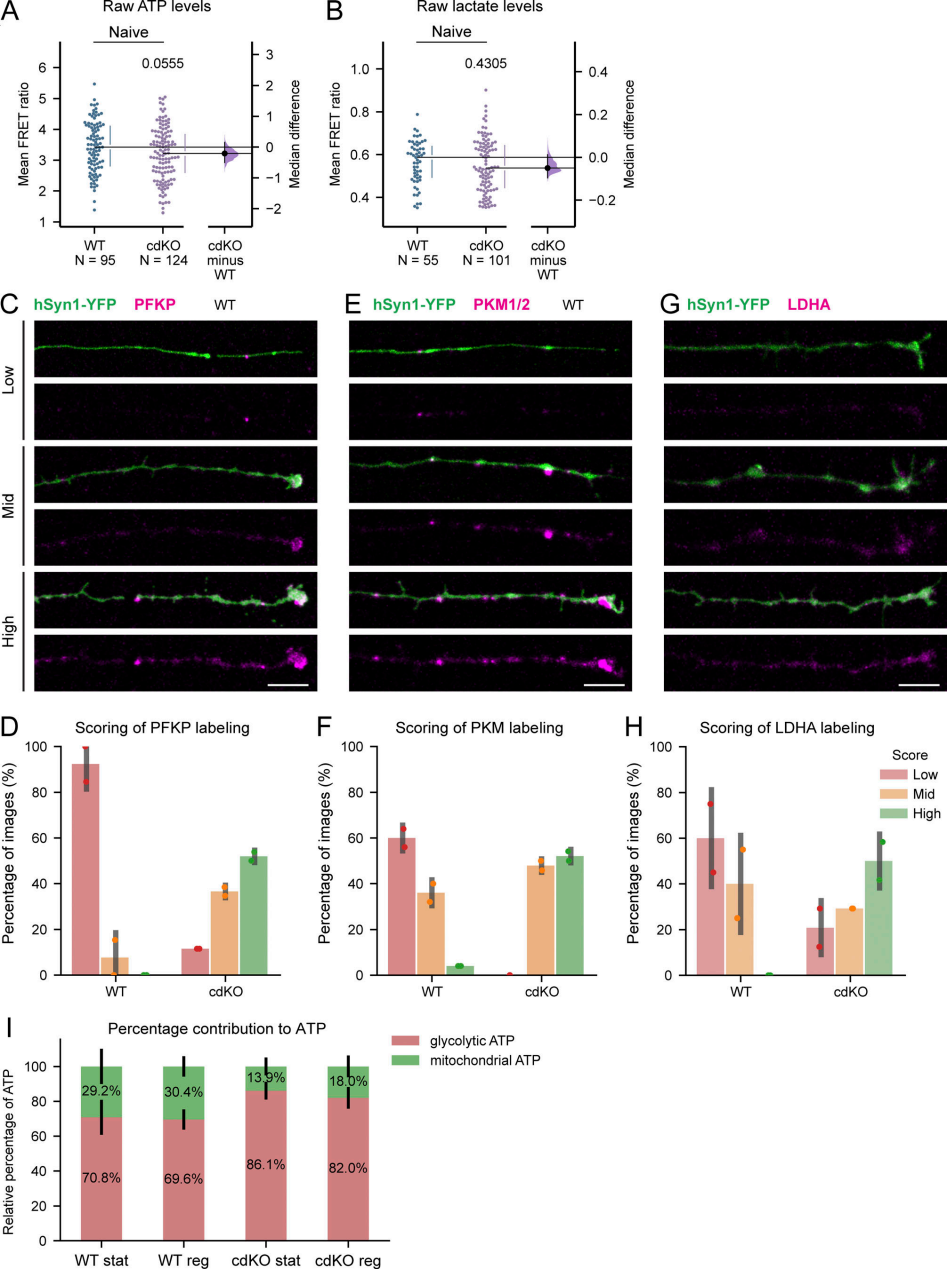
**Deletion of *Pten* and *Socs3* increases axonal glycolytic energy after injury. (A)** Comparison of non-normalized axonal ATP measurements does not identify any significant difference in the intra-axonal concentration of ATP in naive axons between WT and cdKO neurons. **(B)** Comparison of non-normalized axonal lactate measurements does not identify any significant difference in the intra-axonal concentration of lactate in naive axons between WT and cdKO neurons. **(C)** Representative images of axons, after PFKP (Phosphofructokinase, Platelet) immunolabeling (magenta), assigned to each different scoring category. Axons with no appreciable labeling are classified as low. Faint labeling is scored as mid, while bright labeling and the presence of foci are scored as high. Imaging conditions and histogram levels were maintained constant. The reporter hSyn-YFP (green) was encoded by AAV2/2-hSyn1-Laconic-WPRE-hGHp; see Materials and methods. Scale bar 10 µm (**D)** Semiquantitative analysis via randomized blind scoring of PFKP immunolabeled axons by two independent evaluators reveals increased labeling in regenerating cdKO axons as compared to WT ones. *n* > 20 regenerating axons for both genotypes. **(E)** Representative images for each scoring category of PKM (Pyruvate Kinase M1/2) immunolabeled axons (magenta). Axons with faint signal are classified as low. Labeling with sparse foci is classified as mid, while bright labeling and the presence of abundant foci is scored as high. Imaging conditions and histogram levels were maintained constant. The reporter hSyn-YFP (green) was encoded by AAV2/2-hSyn1-Laconic-WPRE-hGHp. Scale bar 10 µm. **(F)** Semiquantitative analysis via randomized blind scoring of PKM immunolabeled axons by two independent evaluators shows increased labeling in regenerating cdKO axons as compared with WT ones. *n* > 20 regenerating axons for both genotypes. **(G)** Representative images of axons, after LDHA (Lactate Dehydrogenase A) immunolabeling (magenta), were assigned to each different scoring category. Axons are classified from low to high based on brightness and uniformity of the labeling along the distal end of the axon. Imaging conditions and histogram levels were maintained constant. The reporter hSyn-YFP (green) was encoded by AAV2/2-hSyn1-Laconic-WPRE-hGHp. Scale bar 10 µm. **(H)** Semiquantitative analysis via randomized blind scoring by two independent evaluators reveals increased LDHA labeling in regenerating cdKO axons as compared with WT ones. *n* > 20 regenerating axons for both genotypes. **(I)** Relative percentage representation of mitochondrial ATP (oligomycin sensitive, green, top) and glycolytic ATP (oligomycin and 2-deoxyglucose sensitive, red, bottom) at 1 dpi. Both static and regenerating cdKO axons show a higher relative percentage of glycolytic ATP as compared to WT axons. The percentage value was obtained by subtracting the baseline measurement in the presence of both 10 µM oligomycin and 50 mM 2-deoxyglucose from the measurements in normal medium and oligomycin-only. Then, the oligomycin-only measurement was expressed as a percentage of the measurement in normal medium. Data from five (A), four (B and I) independent experiments, presented as median ± 25–75th confidence interval (A and B), mean ± SD (D–H), or mean ± SEM (I) and bootstrap 95% confidence interval versus WT (A and B). Mann–Whitney U test (A and B). P values are reported within the graphs.

To determine whether the differences in mitochondrial phenotype between static and regenerating axons were reflected in the axonal energy levels, we expressed the ATeam1.03^YEMK^ biosensor (*AAV2/2-hSyn1-ATeam*^*YEMK*^*-WPRE-hGHp*). The latter undergoes shifts in the ratio of YFP/CFP emission, which are proportional to the ATP concentration, allowing us to measure energy levels within the farthest end of the axon (Fig. 6 A) (Imamura et al., 2009). Consistent with previous studies (Huang et al., 2021), injury caused a significant decrease in axonal ATP at 0 dpi, in both WT and cdKO axons (Fig. 6, B and C). At 1 dpi, in WT RGCs, only regenerating axons restored ATP to levels comparable with naive. On the other hand, at 1 dpi, both static and regenerating cdKO axons restored their ATP concentration to naive levels, with regenerating cdKO axons showing an even higher concentration of ATP compared with uninjured axons (Fig. 6, B and C). Overall, cdKO axons revealed higher ATP levels compared with WT axons after injury, both at 0 and 1 dpi in static and regenerating axons (Fig. 6, B and D). No significant difference in ATP was measured between WT and cdKO uninjured axons (Fig. S4 A).

Considering that all surviving cdKO axonal classes were able to restore ATP levels at 1 dpi (Fig. 6 C), including static axons presenting an altered mitochondrial phenotype, we hypothesized that the deletion of *Pten* and *Socs3* might lead to an increase in non-mitochondrial energy production within the distal axon. Under the Warburg effect (Heiden et al., 2009; Liberti and Locasale, 2016), aerobic glycolysis produces two ATP molecules in the cytosol during the conversion of glucose into lactate (Fig. 6 E). While less efficient than oxidative phosphorylation, it has a faster turnover (Heiden et al., 2009). To assess whether mitochondria-independent energy production takes place in cdKO axons, we expressed the biosensor Laconic (*AAV2/2-hSyn1-Laconic-WPRE-hGHp*) and measured the axonal concentration of lactate (Fig. 6 F). Uninjured axons showed a comparable concentration of lactate in WT and cdKO neurons (Fig. S4 B). We did not detect any significant change in axonal lactate in WT axons at 0 and 1 dpi as compared with naive (Fig. 6 G). On the contrary, cdKO axons significantly upregulated lactate production after injury at 0 dpi. This increase was maintained at 1 dpi in static axons and, strikingly, also in cdKO regenerating axons (Fig. 6 G). In these, the concentration of lactate was higher than that in regenerating WT axons (Fig. 6 H).

**Figure 7.**
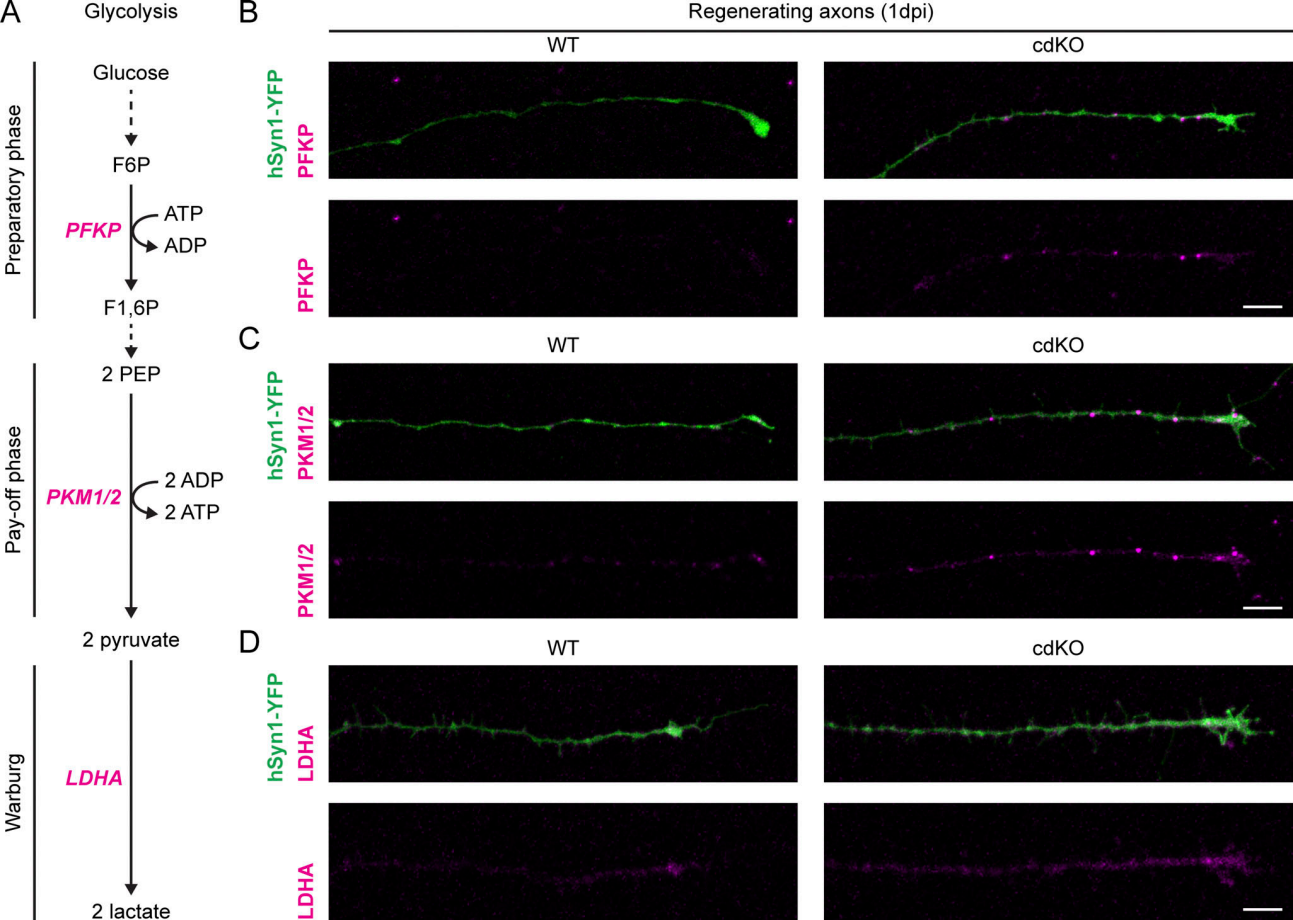
**Regenerating cdKO axons are characterized by enhanced localization of glycolytic enzymes. (A)** Schematic representation of the glycolytic pathway. Glucose is first processed consuming ATP in the preparatory phase, of which PFKP is rate-limiting. During the pay-off phase, per molecule of glucose, two pyruvate molecules are generated, yielding two ATP per each consumed. The rate of this second half of the pathway is controlled by PKM. Finally, when glycolysis is uncoupled from mitochondrial oxidative phosphorylation, pyruvate is reduced by LDHA to lactate, in what is known as the Warburg effect. **(B)** Representative images of PFKB immunolabeling at 1 dpi in regenerating axons of both the WT and cdKO genotype. As compared with WT axons, cdKO ones show increased labeling, characterized by bright foci throughout the distal axonal shaft. Scale bar 10 µm. **(C)** Representative images of PKM immunolabeling (isoform 1 and 2) at 1 dpi in regenerating axons of both the WT and cdKO genotype. Again, cdKO axons reveal a higher degree of labeling as compared with WT ones, with bright foci throughout the distal shaft and at the growth cone. Scale bar 10 µm. **(D)** Representative images of LDHA immunolabeling at 1 dpi in regenerating axons of both the WT and cdKO genotype. cdKO axons display brighter labeling as compared with WT ones, diffused through the growth cone and the distal shaft of the axon. Scale bar 10 µm. The reporter hSyn-YFP was encoded by AAV2/2-hSyn1-Laconic-WPRE-hGHp; see Materials and methods. Steps of the pathway that were omitted are represented by a dotted line. F6P (fructose-6-phosphate), F1,6P (fructose-1,6-bisphosphate), and PEP (phosphoenolpyruvate).

To address whether this increased flux is driven by the allocation of glycolytic enzymes in the distal axon, we labeled them at 1 dpi via immunochemistry. Specifically, we labeled (1) PFKP, which we identified in the transcriptomic study and that controls the rate of the ATP-consuming preparatory phase of glycolysis, (2) pyruvate kinase (PK, isoforms M1 and M2), which dictates the rate of the ATP-producing pay-off phase, and (3) lactate dehydrogenase A (LDHA), which reduces pyruvate into lactate under the Warburg effect (Fig. 7, A–D). For all three enzymes, randomized blind qualitative assessment of immunolabeling in the leading edge revealed an increased localization at 1 dpi in cdKO regenerating axons as compared with WT ones (Fig. S4, C–H). In summary, deletion of *Pten* and *Socs3* improves the restoration of axonal ATP levels after injury and during initiation of regrowth via upregulated glycolysis, likely driven by augmented localization of glycolytic enzymes at the leading edge of the axon.

### Upregulation of glycolysis in cdKO axons is local and serves as the primary provider of ATP

**Figure 8.**
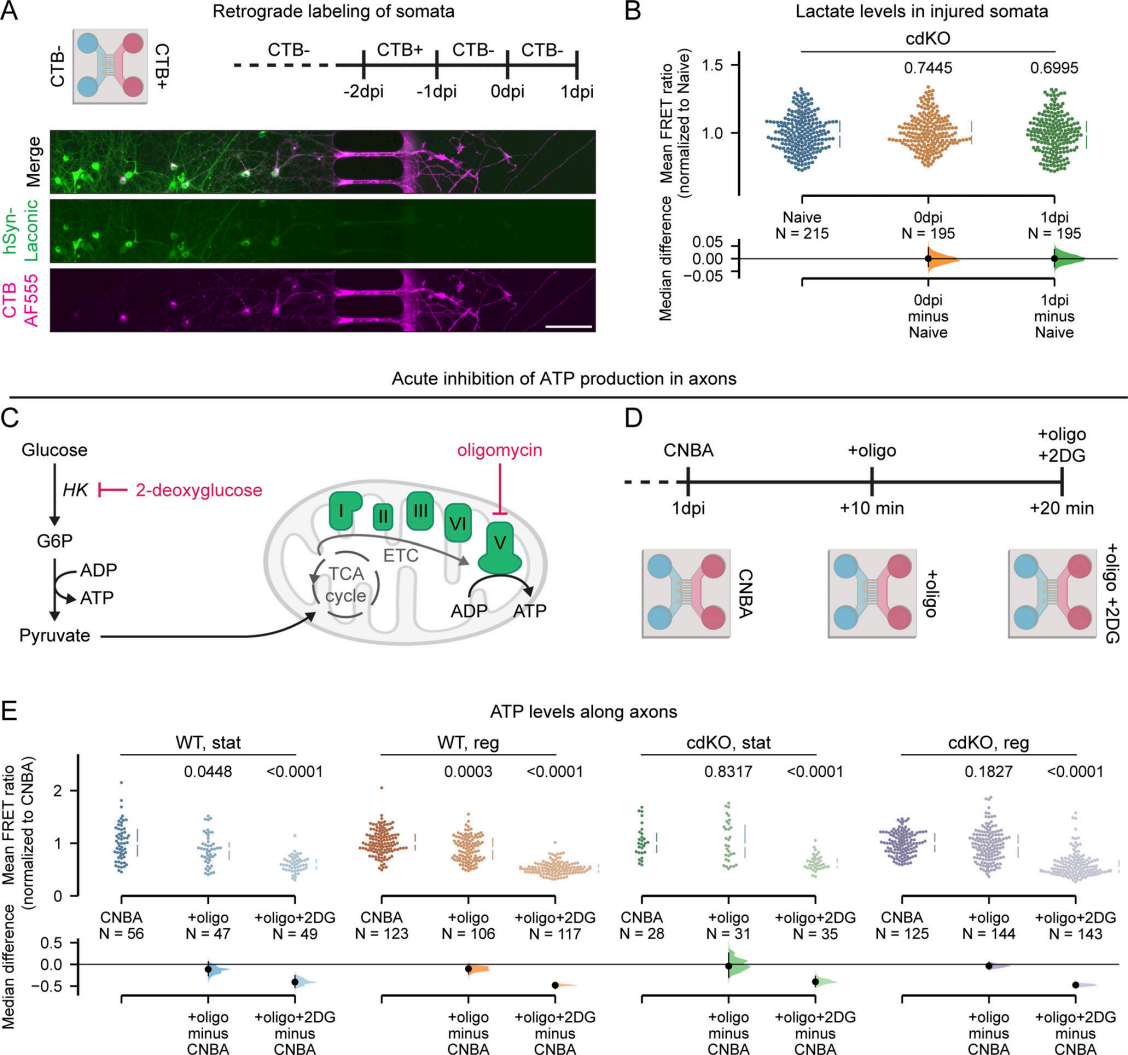
**The upregulation of glycolysis in cdKO axons is local and is the primary provider of ATP. (A)** Schematic representation of retrograde labeling of microgroove-crossing neurons with CTB, allowing to specifically label the somata of the RGCs that will be injured. Scale bar 100 µm. **(B)** Quantification of intracellular lactate within the somata of cdKO injured RGCs shows no upregulation of aerobic glycolysis in the somatic compartment immediately after injury (0 dpi) and at 1 dpi. **(C)** Graphical representation of the mechanism of metabolic inhibition of oligo and 2DG. Oligo is an inhibitor of the mitochondrial ATP synthase (or Complex V), which is the last step of the electron transport chain (ETC), responsible for producing ATP from the activity of the tricarboxylic acid cycle (TCA or Krebs cycle). 2DG is a strong competitive inhibitor of hexokinase (HK) activity, leading to the complete blockade of the entire glycolytic pathway. **(D)** Schematic representation of the sequential acute inhibition of oxidative phosphorylation and glycolysis, via administration in complete Neurobasal-A (CNBA) of 10 µM oligo and of 50 mM 2-DG, respectively, in the axonal compartment. This first removes the mitochondrial component of ATP production and subsequently abolishes the glycolytic one, allowing us to determine their relative contribution to the total axonal ATP pool. **(E)** Quantification of the FRET ratio along axons in normal CNBA and in presence of oligo (+oligo) or oligo and 2-DG (+oligo+2DG) for each axon class. A significant decrease in ATP concentration is detected in WT axons after inhibition of oxidative phosphorylation, but not in cdKO axons. Data from four independent experiments are presented as median ± 25–75th confidence interval and bootstrap 95% confidence interval versus relative naive (B) or relative CNBA (E). Kruskal–Wallis ANOVA. P values are reported within the graphs.

To further determine whether the upregulation of glycolysis in cdKO neurons is a local compensatory effect within the axons rather than a neuron-wide phenotype, we measured the concentration of lactate within the soma of the injured RGCs. To achieve this, prior to injury, we retrogradely traced the RGCs of which the axons extend into the axonal channel and thus that will be injured during axotomy (Fig. 8 A). Strikingly, no somatic upregulation of lactate production was detected in injured RGCs at both 0 and 1 dpi as compared with naive (Fig. 8 B), suggesting that the upregulation of aerobic glycolysis is a local axon-restricted effect.

Finally, to determine the relative contribution of oxidative phosphorylation and glycolysis to the pool of axonal ATP at 1 dpi, we sequentially inhibited both processes in the axonal compartment with the mitochondrial ATP synthase inhibitor oligomycin (oligo) and the strong competitive inhibitor of hexokinase, 2-deoxyglucose (2DG), respectively (Fig. 8, C and D) (Connolly et al., 2018; Wick et al., 1957). In WT axons, we detected a significant reduction in ATP after inhibition of oxidative phosphorylation and a further reduction upon additional inhibition of glycolysis (Fig. 8 E). On the other hand, in dKO axons, we did not observe a significant decrease in ATP with oligomycin, but rather only after combined inhibition of glycolysis (Fig. 8 E). This suggests that cdKO axons can sustain ATP via glycolysis alone. The relative percentages of ATP production calculated from this experiment are reported in the supplement (Fig. S4 I). Together, these data revealed that the cdKO axons undergo a local upregulation of glycolysis after injury and that this provides the bulk of the axonal ATP.

### Downregulation of axonal glycolysis reverses the regeneration phenotype upon *Pten* and *Socs3* co-deletion

**Figure 9.**
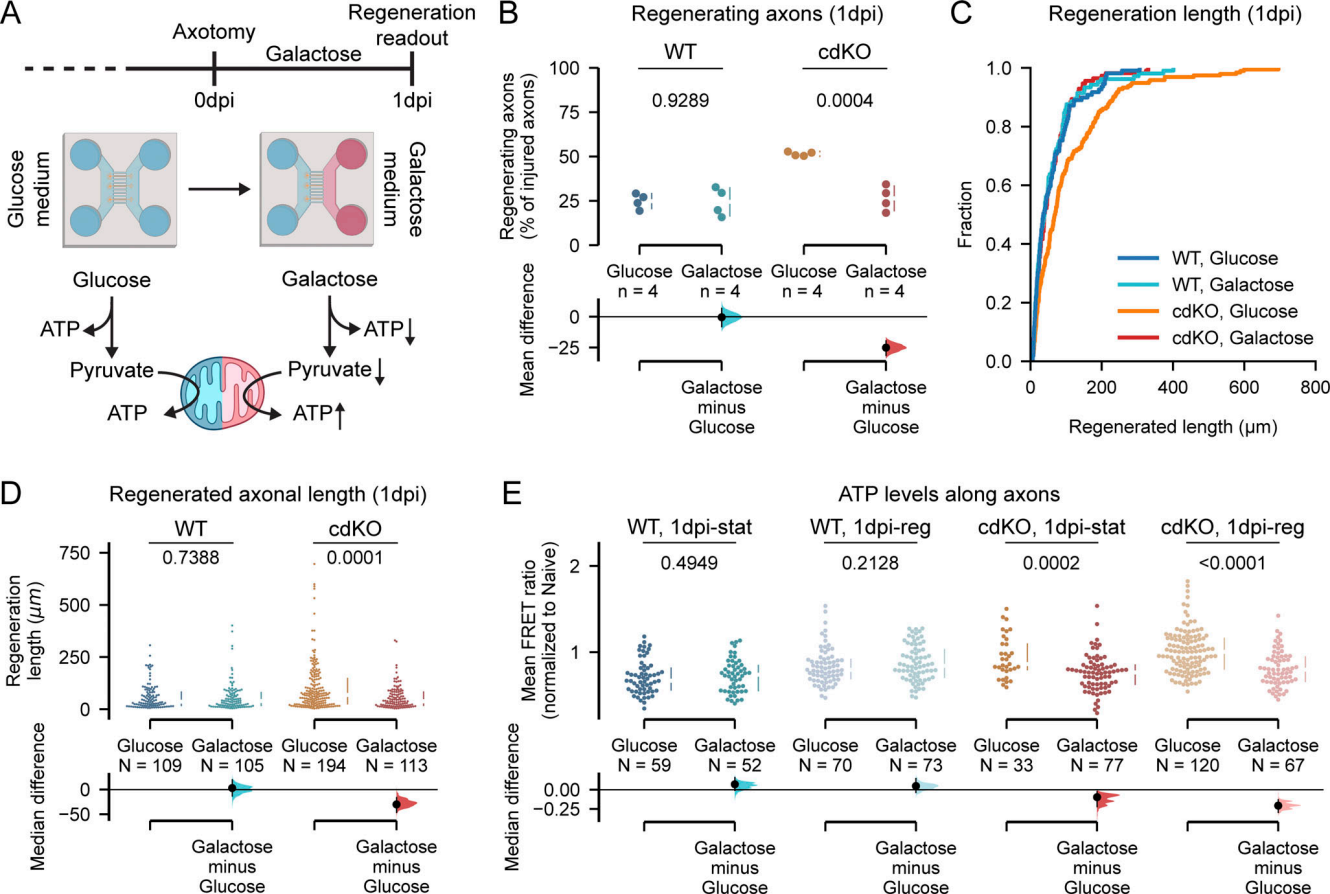
**Impairment of axonal glycolysis abolishes the regeneration phenotype of cdKO axons, via decreased axonal ATP. (A)** Schematic representation of the axonal glycolysis downregulation. Glucose in the medium is switched to galactose after axotomy and maintained until 1 dpi. Galactose is processed via glycolysis at a slower rate, leading to reduced glycolytic ATP and a relative induction of mitochondrial ATP. **(B)** Quantification of the percentage of regenerating axons shows that galactose treatment does not affect WT axons but reverses the enhanced regeneration phenotype of cdKO axons. **(C)** Cumulative distribution of axonal lengths reveals that galactose treatment reduces the regrowth length in cdKO axons at 1 dpi. **(D)** Quantification of the regenerated axonal length shows that galactose treatment induces a significant decrease in the average axonal regenerated length in cdKO axons at 1 dpi. **(E)** Quantification of ATP levels along axons (ATeam biosensor) reveals that downregulation of axonal glycolysis does not impact the ATP concentration of WT axons at 1 dpi but reduces the ATP concentration of both static and regenerating cdKO axons. Data from four independent experiments are presented as mean ± SD (B) or median ± 25–75th confidence interval (D and E) and bootstrap 95% confidence interval versus glucose. Student’s *t* test (B), Mann–Whitney U test (D and E). P values are reported within the graphs.

**Figure S5.**
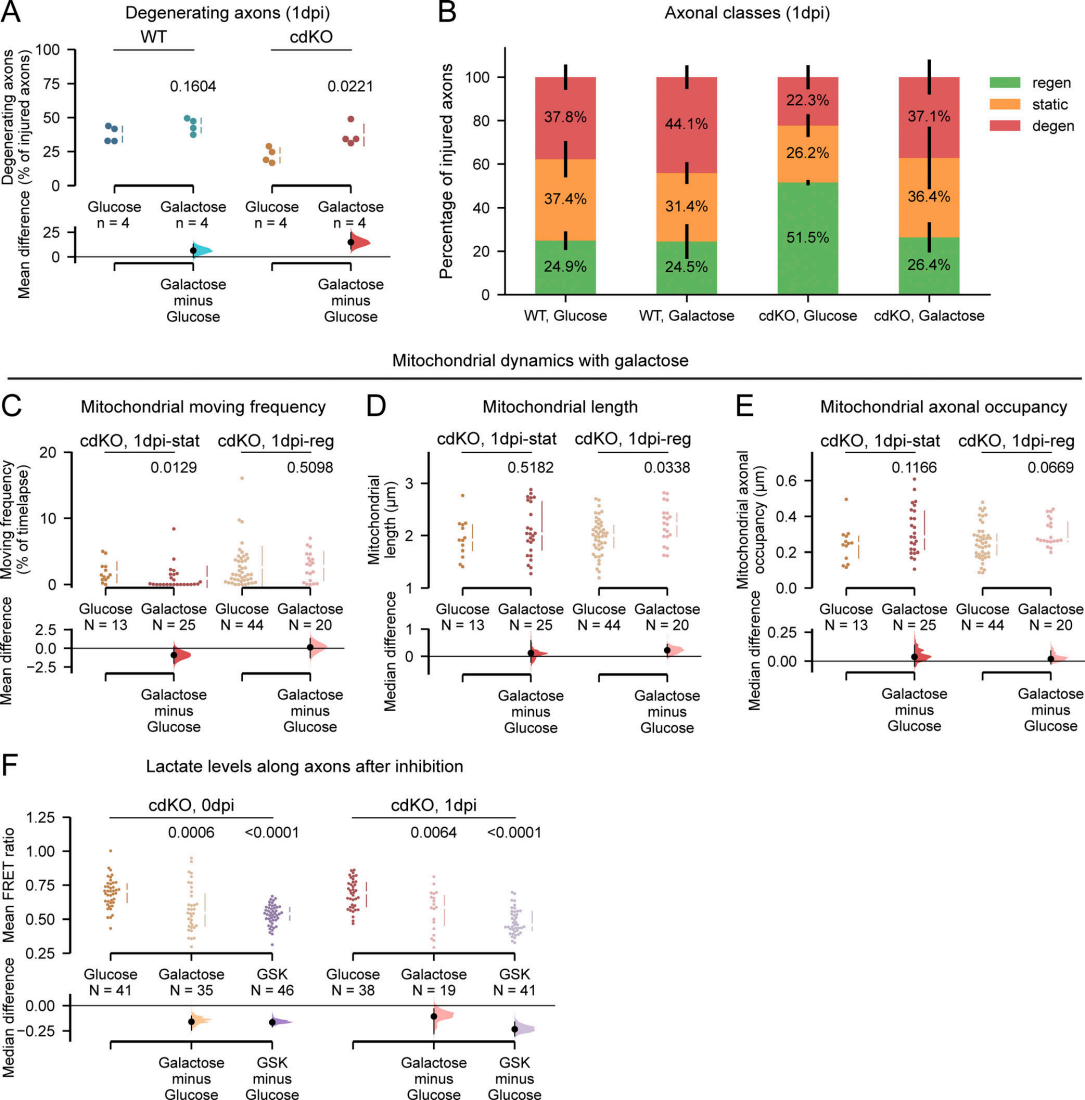
**Downregulation of axonal glycolysis reverses the regeneration phenotype of *Pten* and *Socs3* co-deleted neurons. (A)** Quantification of the percentage of degenerating axons at 1 dpi shows that galactose treatment does not affect WT axons but reverses the reduced degeneration phenotype of cdKO axons. **(B)** Combined graphical representation of the percentages of degenerating (red, top), static (orange, middle) and regenerating (green, bottom) axons for WT and cdKO neurons at 1 dpi. Galactose treatment reverses the phenotype of cdKO neurons. **(C)** Quantification of mitochondrial moving frequency at 1 dpi shows a minor decrease in mitochondrial transport in static axons cultured in galactose as compared to glucose medium, but no significant decrease in regenerating axons. **(D)** Quantification of mitochondrial length in cdKO axons at 1 dpi shows a minor but significant increase in mitochondrial elongation in regenerating axons when cultured in galactose. This is possibly due to compensatory mitochondrial fusion associated with increased oxidative phosphorylation. **(E)** Quantification of axonal mitochondrial occupancy in cdKO neurons at 1 dpi shows no difference in mitochondrial density between axons cultured in glucose or galactose medium. **(F)** Quantification of intra-axonal lactate concentration at 0 and 1 dpi in normal medium (glucose), in galactose medium and in the presence of the lactate dehydrogenase inhibitor GSK (5 µM). Galactose and GSK media were administered in the axonal compartment at 0 dpi after axotomy and maintained until 1 dpi. Both slowing down glycolysis upstream with galactose as well as inhibiting pyruvate reduction significantly decreased the concentration of lactate in cdKO axons at both time points. Data from four independent experiments, presented as mean ± SD (A–C) or median ± 25–75th confidence interval (D–F) and bootstrap 95% confidence interval versus glucose. Student’s *T* test (A), Mann–Whitney U test (C–E), or Kruskal–Wallis ANOVA (F). P values are reported within the graphs.

To address whether the increased glycolysis upon injury in cdKO axons is necessary for regeneration, we downregulated the glycolytic flux in the axonal compartment following axotomy. This was achieved via the substitution of glucose for galactose in the axonal medium from 0 dpi until 1 dpi (Fig. 9 A). Galactose is an alternative sugar, which can be converted into glucose-6-phosphate (G6P) through the Leloir pathway to feed into glycolysis (Frey, 1996; Li et al., 2023; Robinson et al., 1992). In the absence of glucose, the conversion of galactose into G6P becomes rate-limiting, reducing the flux of glycolysis and reducing glycolytic ATP (Robinson et al., 1992) (Fig. 9 A). With galactose as a substrate, cells have been shown to rely more heavily on mitochondrial oxidative phosphorylation to meet energy demands (Aguer et al., 2011; Robinson et al., 1992). The effectiveness of this approach was verified by measuring the intra-axonal concentration of lactate, which was significantly reduced at 0 and 1 dpi for cdKO axons in galactose medium (Fig. S5 F).

Galactose treatment did not alter the limited regeneration phenotype of WT axons, both in terms of the percentage of regenerating axons (Fig. 9 B) as well as the length (Fig. 9, C and D). On the other hand, the downregulation of axonal glycolysis in cdKO axons completely reversed the induced regeneration phenotype of *Pten* and *Socs3* co-deletion. Both the percentage of regenerating cdKO axons as well as their length significantly decreased to levels comparable with WT axons when grown in galactose medium (Fig. 9, B–D). Also, the percentage of degenerating and static cdKO axons decreased to levels comparable with WT when in a galactose medium (Fig. S5, A and B).

To determine whether the downregulation of glycolysis impacted the intra-axonal energy levels, we measured the concentration of ATP within the distal axon during galactose treatment. Limiting glycolysis did not impact ATP production in both static and regenerating WT axons at 1 dpi (Fig. 9 E). On the other hand, both static and regenerating cdKO axons showed significantly lower ATP levels at 1 dpi in galactose medium as compared with glucose (Fig. 9 E). Finally, we labeled mitochondria with MitoTracker to assess whether the treatment affected the mitochondrial phenotype. Notably, galactose did not alter mitochondrial transport and density in cdKO axons (Fig. S5, C and E). At 1 dpi, we measured comparable mitochondrial trafficking between regenerating axons in the glucose and galactose conditions (Fig. S5 C). Only a minor increase in mitochondrial length was measured in regenerating axons when grown in galactose medium (Fig. S5 D). This is likely due to a compensatory increase in mitochondrial fusion to sustain oxidative phosphorylation. In summary, these findings support the hypothesis that local upregulation of axonal glycolysis underlies the enhanced regrowth of axons upon deletion of *Pten* and *Socs3*.

### Lactate production is required for enhanced axonal survival and regrowth initiation but is dispensable for elongation

**Figure 10.**
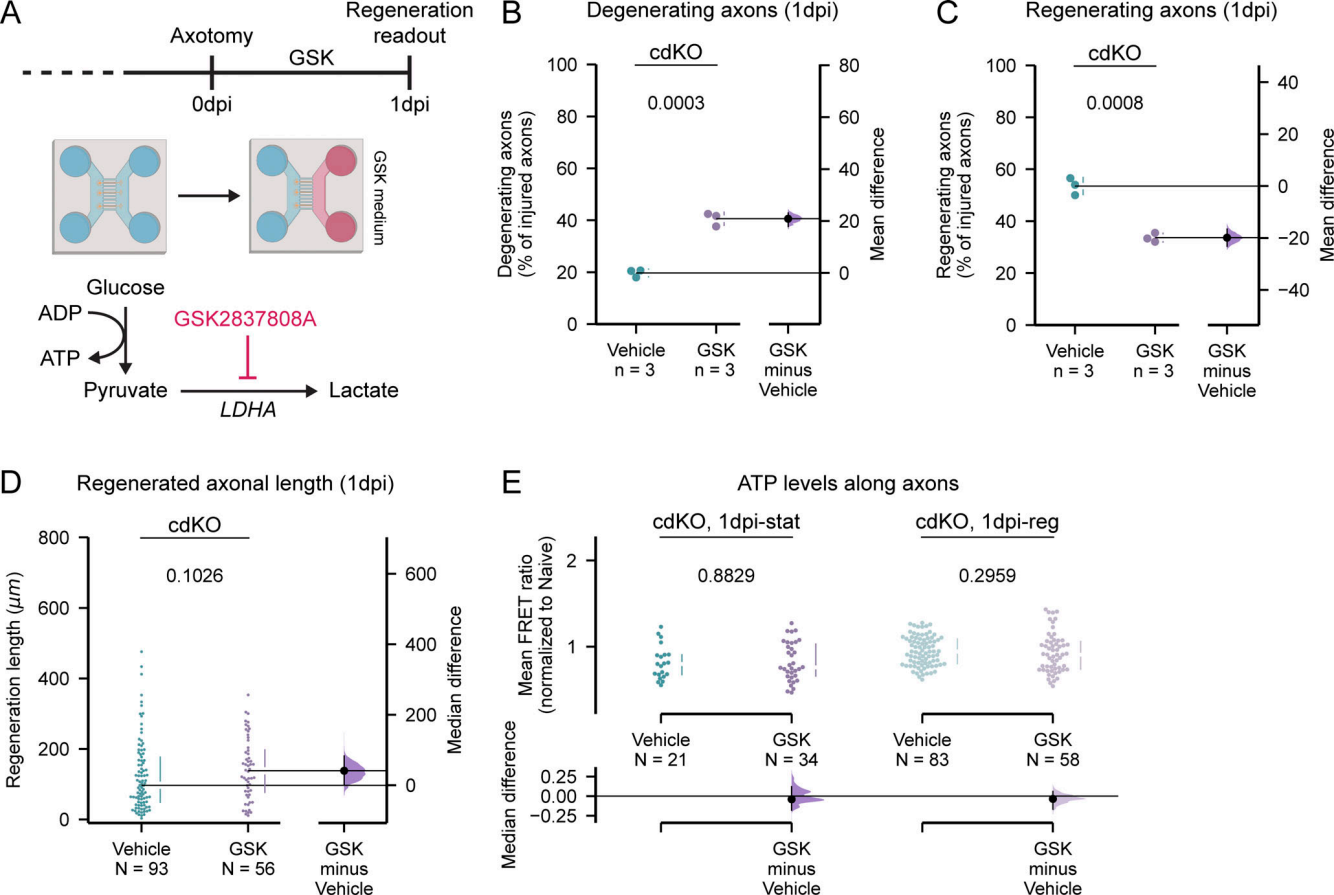
**Lactate production is required for enhanced axonal survival and regrowth initiation but is dispensable for elongation. (A)** Schematic representation of the axonal lactate production inhibition in cdKO RGCs. The LDH inhibitor GSK (5 µM) was administered on the axonal compartment following injury and until 1 dpi. **(B)** Quantification of the percentage of degenerating axons reveals that lactate production inhibition increases the extent of axonal degeneration of cdKO axons at 1 dpi. **(C)** Quantification of the percentage of regenerating axons shows that lactate production inhibition reverses the enhanced regeneration phenotype of cdKO axons at 1 dpi. **(D)** Quantification of the regenerated axonal length reveals that lactate production inhibition does not impact the average axonal regenerated length in cdKO axons at 1 dpi. **(E)** Quantification of the FRET ratio along axons reveals that lactate production inhibition does not impact the ATP concentration in both static and regenerating cdKO axons at 1 dpi. Data from three independent experiments are presented as mean ± SD (B and C) or median ± 25–75th confidence interval (D and E) and bootstrap 95% confidence interval versus vehicle. Student’s *t* test (B and C), Mann–Whitney U test (D and E). P values are reported within the graphs.

Upstream downregulation of axonal glycolysis via galactose treatment impaired both axonal survival and axonal regeneration in cdKO RGCs. As lactate production is immediately upregulated after injury in cdKO axons in vitro and since we observed a transcriptomic signature of lactate export only in the early phases of axonal regeneration in vivo, we hypothesized that it might specifically be a mechanism of axonal survival and possibly initiation rather than axonal elongation. To test this, we inhibited lactate production locally in the axonal compartment after injury via administration of the LDHA inhibitor GSK2837808A (GSK) (Fig. 10 A). GSK prevents the reduction of pyruvate into lactate, effectively inhibiting the Warburg effect (Fig. 10 A) (Iwata et al., 2023; Billiard et al., 2013), and accordingly, we measured a significantly reduced intra-axonal concentration of lactate in the presence of GSK in cdKO axons at 0 and 1 dpi (Fig. S5 F). Axonal inhibition of pyruvate reduction doubled the percentage of degenerating axons at 1 dpi as compared with the vehicle treatment (Fig. 10 B) to a level comparable with galactose and WT RGCs. Moreover, GSK treatment significantly reduced the number of regenerating axons as compared with vehicle treatment (Fig. 10 C). Together these data highlight the important role of lactate production during injury response, prevention of degeneration, and initiation of axonal regrowth. On the other hand, contrary to galactose, GSK treatment did not impair the regrowth length of the few regenerating axons (Fig. 10 D). Finally, we assessed whether GSK treatment would affect ATP production within the axons at 1 dpi with the Förster resonance energy transfer (FRET) biosensor ATeam. Unlike upstream downregulation with galactose, the inhibition of lactate production did not alter the concentration of ATP within the distal end of both the static and regenerating axons (Fig. 10 E). Taken together, these findings show that lactate production is required to induce the axonal regeneration phenotype characteristic of cdKO RGCs. The mechanism though seems to be related to axonal survival and regrowth initiation rather than axonal elongation.

## Discussion

In this study, using transcriptomic data from Jacobi et al. (2022), we identified an upregulated expression of glycolytic genes after optic nerve injury in *Pten* and *Socs3* co-deleted RGCs. Using a culture of RGCs in microfluidic devices, we demonstrated that axotomy-induced axonal regeneration is characterized by the restoration of mitochondrial axonal transport and that the increased regeneration upon *Pten* and *Socs3* deletion is accompanied by an upregulated mitochondrial trafficking after injury. Nevertheless, we demonstrated that local glycolysis significantly contributes to energy production in the distal section of these regenerating axons and that restricting glycolysis with galactose reversed their regeneration capabilities in terms of both axonal number and regrowth length. On the other hand, blocking lactate production in cdKO RGCs decreased the number of regenerating axons without affecting the regrowth length, suggesting the importance of lactate for injury survival specifically as opposed to the broader role of glycolysis in all phases of axonal regeneration.

### Circumventing an irreversible energy crisis

Injury has been reported to alter the axonal environment, leading to mitochondrial depolarization and dysfunction (Zhou et al., 2016; Petrova et al., 2021). This causes a depletion of axonal ATP, which is often associated with Wallerian degeneration (Hopkins et al., 2021; Conforti et al., 2014). Unfortunately, mitochondrial transport is progressively decreased during neuronal maturation, with increased mitochondrial docking at the site of synapses, mediated by the adaptor proteins connecting the mitochondria to the kinesin and dynein motors, such as Miro and syntaphilin (Lewis et al., 2016; Cheng et al., 2022; Petrova et al., 2021). The inability to remobilize mitochondria has been ascribed as one of the mechanisms restricting axonal regeneration in the mammalian CNS (Cheng et al., 2022; Petrova et al., 2021).

In accordance, with our setup of cultured RGCs, we have shown that restoration of axonal mitochondrial transport and morphology is indeed required for axotomy-induced axonal regrowth. Unlike a previous report by Cartoni et al. on embryonic cortical neurons (Cartoni et al., 2017), we did not detect a higher basal mitochondrial transport in uninjured axons upon *Pten* and *Socs3* co-deletion. Rather, the enhanced trafficking in cdKO RGCs as compared with WT ones was detected only at 1 dpi in regenerating axons. This difference observed in uninjured axons might be due to a general difference between the neuronal classes and experimental design. Strikingly, the enhanced regeneration associated with *Pten* and *Socs3* deletion in our retinal neurons is accompanied by an immediate upregulation of axonal lactate-producing aerobic glycolysis, preceding the detected increase in mitochondrial transport. Inhibition of lactate production led to decreased axonal survival and regrowth, suggesting that energy production via aerobic glycolysis is essential to overcome the axonal injury. Nonetheless, we observed that also in regenerating cdKO axons, during elongation, a considerable fraction of energy is derived from glycolysis and that limiting the glycolytic rate impairs axonal ATP production and axonal regrowth. These findings are supported by the in vivo transcriptomic evidence of increased glycolysis, including all rate-limiting steps and the lactate exporter MCT4, after optic nerve injury in cdKO RGCs. Thus, enhanced glycolysis, alongside restored mitochondrial function, is essential for the regeneration phenotype characteristic of cdKO axons.

### Glycolysis: An essential gear in the axonal machinery

Neurons predominantly rely on mitochondrial metabolism to meet energy demands. Indeed, the differentiation of neuronal progenitor cells into neurons is accompanied by a shift from glycolysis to oxidative phosphorylation (Zheng et al., 2016; Iwata et al., 2023). Metabolic support from glycolytic glial cells, i.e., astrocytes and oligodendrocytes, involves lactate production that is taken up by neurons, converted to pyruvate, and used for oxidative phosphorylation (Bonvento and Bolaños, 2021; Chamberlain and Sheng, 2019). Lately, researchers started to challenge this notion, showing that glucose uptake and glycolysis are essential for basal neuronal functions (Li et al., 2023; Wei et al., 2023). Recent evidence in *C. elegans* suggests that neurons autonomously carry out glycolysis and can regulate it at a subcellular compartment level (Wolfe et al., 2024). Neuronal stimulation has been reported to induce glycolysis rather than oxidative phosphorylation in vivo in mice, temporarily making neurons net exporters of lactate (Díaz-García et al., 2017). Moreover, fast axonal transport has been described to be fueled by the glycolytic metabolon assembled on the vesicles rather than by mitochondria (Zala et al., 2013; Hinckelmann et al., 2016), and this requires the reduction of pyruvate into lactate (Mc Cluskey et al., 2023). Likewise, both developmental axonal growth and retraction in vitro have been shown to rely on glycolysis in embryonic chick dorsal root ganglia (DRG) and embryonic cortical rat neurons, respectively (Ketschek et al., 2021; Santos et al., 2023). Given the short half-life and low diffusion rate of ATP, processes like cytoskeletal remodeling require ATP produced locally by tightly coupled glycolytic activity (Hubley et al., 1996; Ketschek et al., 2021; Santos et al., 2023). This might explain the beneficial role of glycolytic ATP in cdKO axons after injury, facilitating cytoskeletal reorganization during growth-cone formation and fueling vesicular transport of somatic material into the axon.

### A strengthened glycolysis-mitochondria axis

In the context of axonal injury, the ability to upregulate glycolysis might thus fill the energy gap left by depolarized and dysfunctional mitochondria, counteracting degeneration, and initiating regrowth. In the PNS, a recent study reported that glycolysis is activated in the axons of the rat sciatic nerve after injury and that inhibition of glycolysis increased Wallerian degeneration (Takenaka et al., 2023). Moreover, it has been demonstrated that the activity of the hypoxia-inducible factor (HIF1α), which has many glycolytic genes as a targets, plays a crucial role in the regeneration of murine DRGs (Cho et al., 2015). These data support our observations in regeneration-competent CNS neurons, showing injury-induced upregulation of glycolytic genes in *Pten* and *Socs3* co-deleted RGCs in vivo. Furthermore, we have demonstrated that this upregulation is local to the axons and that lactate production is required to enhance survival and the initiation of regrowth in cdKO axons. This mechanism might be related to the restoration of NAD^+^, as we detected an increase in the expression of rate-limiting enzymes of the NAD salvage pathway in vivo. Indeed, depletion of NAD^+^ is a hallmark of Wallerian degeneration (Conforti et al., 2014; Hopkins et al., 2021).

The role of glycolysis, though, is critical beyond the injury response phase as we have shown that also in regrowing, elongating axons, the majority of axonal ATP is glycolytic rather than derived from mitochondria. Nonetheless, in this scenario of upregulated glycolysis, mitochondria are likely not redundant as they exert a wide array of cellular functions besides producing ATP. Their primary role in this context might rather lie within these “secondary” functions, which include calcium buffering, redox homeostasis, and the generation of precursors for the synthesis of macromolecules essential for cellular survival (Spinelli and Haigis, 2018; Rangaraju et al., 2019). While the latter has not been studied as well in neurons as in other cell types, it has been shown that neuronal lipid synthesis requires cooperation between mitochondria and the endoplasmic reticulum (Tracey et al., 2018; Rangaraju et al., 2019). In proliferating cells, under the Warburg effect, while aerobic glycolysis provides most of the ATP (Liberti and Locasale, 2016; Heiden et al., 2009), mitochondria are still functional, and via the Krebs cycle, they have a critical role in providing acetyl-CoA for fatty acid synthesis and aspartate for amino acid synthesis. Neurons could also benefit from this separation of metabolic roles during axonal elongation, optimizing both energy production via glycolysis and the synthesis of macromolecules necessary for axonal growth via mitochondria. As such, the integration of a high glycolytic flux with the maintenance of healthy mitochondria, as seen in regenerating cdKO axons, could provide an ideal biochemical environment for regrowth.

Anabolic pathways often require copious amounts of NADPH, which are restored via reduction of NADP^+^ by the pentose phosphate pathway (PPP). While we did not observe any upregulation of the rate-limiting enzymes of the oxidative branch of the PPP, we specifically detected an increased expression of *Taldo1*, the rate-limiting step of the non-oxidative branch. In postmitotic cells like neurons, which do not require pentose for nucleotide synthesis, this branch converts excess ribulose-6-phosphate, generated while reducing NADPH, into glycolysis intermediates (TeSlaa et al., 2023). Glycolysis and the PPP, utilizing both G6P, are generally competing pathways. In this scenario, increased transaldolase activity might allow NADPH generation while still returning glucose-derived intermediates to glycolysis to be used for ATP generation. Nevertheless, follow-up research is required to elucidate the role of this specific transcriptional upregulation.

### Limitations of the study

The functional experiments of this study were conducted in an in vitro setting, which can cause adaptations to basal neuronal metabolism as compared with in vivo conditions. First, we make use of cells isolated from postnatal day 2–3 (P2–3) mouse pups. Although the RGCs approximated mature phenotypes after 2 wk in culture, they might not fully recapitulate an adult phenotype. Second, while we employed mixed cultures containing both retinal neurons and glia, the latter was absent from the axonal compartment, preventing possible axon–glia metabolic interactions that could occur in vivo. Schwann cells have been shown to support regeneration in the PNS via increased glycolytic activity and lactate shuttle to the axons (Li et al., 2020). Conversely, in the context of optic nerve crush, resident oligodendrocytes were shown to undergo demyelination and partial cell death (Xing et al., 2023). How this disrupted environment affects the metabolic support of injured and regrowing axons is unclear. During the first phases of regeneration, it is reasonable to speculate that the leading edges of axons rely primarily on intrinsic glucose metabolism, as supported by the transcriptomic evidence of increased glycolytic gene expression in cdKO RGCs.

Culture media optimized for neuronal growth, such as Neurobasal-A, often contain concentrations of glucose which are hyperphysiological. Not much is known about how this affects neuronal metabolism, but the use of high-glucose media has been shown to induce a lower reliance on mitochondrial metabolism in yeast and mammalian cell lines, in what is known as the Crabtree effect (Crabtree, 1935). Nonetheless, we detected an increase in lactate production and glycolytic flux after injury specifically in axons rather than somas and in *Pten* and *Socs3* co-deleted neurons rather than WT ones. This aligns with the enhanced localization of glycolytic enzymes observed in these axons and the transcriptional signature in vivo of the crushed cdKO RGCs, supporting the hypothesis of enhanced glycolysis being a specific injury-induced mechanism underlying axonal regeneration.

### Impact and future perspectives

While the deletion of *Pten* and *Socs3* affects a wide array of cellular functions and is not suited for clinical translation, the targeting of glycolysis could support and be synergistic with other less invasive strategies to induce axonal regrowth, such as targeting of interleukin 6 signaling or the overexpression of protrudin (Petrova et al., 2020; Leibinger et al., 2013b). Selective pharmacological targeting and upregulation of glycolysis is challenging, as most of the drugs so far have been developed to inhibit it in the context of cancer research. A potential way to induce glucose uptake and utilization is via metformin treatment (Blumrich and Dringen, 2019). This could prove beneficial for neuronal survival but presents numerous side effects, such as increased AMPK signaling (Goel et al., 2022), that could antagonize the mTOR signaling that is believed to be required for axonal regeneration. Moreover, pharmacological treatment is likely not suitable for targeting metabolism at the subcellular level. For this purpose, gene therapy holds the most promise. Viral-mediated overexpression of key regulatory enzymes, such as PFK, PFKFB3, and LDH, fused with axonal targeting sequences, could serve to upregulate glycolysis in injured axons. In summary, the manipulation of glycolysis and axonal metabolism at large in non-genetically modified animals could provide new therapeutic avenues to unlock the regeneration potential of the mammalian CNS.

## Materials and methods

### Animals and housing

Experiments were performed on male and female P2–3 *Pten*^*fl/fl*^*;Socs3*^*fl/fl*^ mice, obtained by crossing *Pten*^*fl/fl*^ and *Socs3*^*fl/fl*^ mice (Yasukawa et al., 2003; Lesche et al., 2002), which were purchased from Jackson Laboratories. For mitochondrial trafficking experiments, male and female C57Bl6 mice were used to generate WT cells (see Viral vector section of the Materials and methods).

Mice were housed in a temperature-, light-, and humidity-controlled environment under a 12-h light/dark cycle and with ad libitum access to food and water. All animal experiments were approved by the Institutional Ethical Committees for Animal Experimentation of KU Leuven and conducted by following strictly the European and Belgian legislation.

### Primary retinal cultures

#### Retinal cell isolation

Retinal cells were isolated from P2–P3 mouse pups following previously reported methods with minor modifications (Pereiro et al., 2018). Retinae were dissected from the eyes in warm sterile Dulbecco PBS (DPBS, Cat# 14190250; Gibco) and collected during the procedure in warm Dulbecco’s Modified Eagle Medium (DMEM, Cat# 31966021; Gibco) supplemented with 50 µg/ml gentamycin (Cat# G1272; Merck) (gDMEM). After dissection, retinae were dissociated with 16 U/ml of papain (Cat# LS003118; Worthington) in 1 ml of gDMEM for 30 min, gently triturated with a pipette tip, filtered through a 40-µm cell strainer and resuspended in 200 μl of complete Neurobasal-A medium (CNBA) (Neurobasal-A [Cat# 10888022; Gibco], 2 mM GlutaMAX [Cat# 35050061; Gibco], 10 mM HEPES [Cat# 15630056; Gibco], 2% B27 [Cat# 0080085SA; Gibco], 5 µM forskolin [Cat# F6886; Merck], 50 µg/ml gentamicin [Cat# G1272; Merck]) with the addition of 45 U/ml of DNAse (Cat# 90083; Thermo Fisher Scientific) to prevent clumping.

#### Microfluidic seeding and culture

Retinal cells were seeded on XC150 microfluidic devices (Cat# XC150; Xona Microfluidics), which were previously sequentially coated with poly-*D*-lysin (0.1 mg/ml in water, Cat# P6407; Merck) and laminin (2 mg/ml in DPBS, Cat# L2020; Merck), both overnight at room temperature. Briefly, 10 μl of medium containing 250,000 cells were added to the somatic channel of the microfluidic device (MFD). Cells were allowed to attach for 1 h and, subsequently, the wells of the somatic chamber of the MFD were filled with somatic CNBA (sCNBA: CNBA, 2 ng/ml CNTF (Cliary Neurotrophic Factor) [Cat# 450-13; PeproTech], 5 ng/ml BDNF (Brain Derived Neurotrophic Factor) [Cat# 450-02; PeproTech]), and on the axonal chamber with axonal CNBA (aCNBA: CNBA, 20 ng/ml CNTF, 50 ng/ml BDNF). A small fluidic gradient was kept across the grooves during normal culture by adding 150 μl of medium per well on the somatic side and 120 μl per well on the axonal one. In each well, half of the medium was replaced with a fresh one every other day during the extent of the culture. Cells were kept in a humidified incubator supplied with 5% CO_2_ at 37°C.

#### Viral constructs and transduction

Retinal cells were transduced on 1DIV with AAV vectors diluted in sCNBA and added to the somatic chamber only. For the imaging of ATP levels, cells were transduced with an *AAV2/2-hSyn1-ATeam*^*YEMK*^*-WPRE-hGHp* vector (Viral Vector Facility, University of Zurich, Zürich, Switzerland, 1.0 × 10^10^ genome copies (GC)/ml final titer in medium) and for the imaging of lactate levels with a *AAV2/2-hSyn1-Laconic-WPRE-hGHp* vector (Viral Vector Facility, University of Zurich, Zürich, Switzerland, 6.7 × 10^9^ GC/ml final titer in medium). Only for the cdKO condition, an *AAV2/2-hSyn1-Cre-t2A-mKate2* vector (Charles River, 1.8 × 10^9^ GC/ml final titer in medium) vector was used in combination with the reporters to induce gene recombination and deletion in *Pten*^*fl/fl*^;*Socs3*^*fl/fl*^ cells. To evaluate the knockout efficiency and axonal regeneration in Figs. 3 and S3, ATeam was used as a fluorescent reporter. In this case, only the YFP fluorophore was excited and is thus reported as hSyn-YFP in the figures and text. The use of a hSyn promoter ensures a high degree of RGC specificity.

For mitochondrial imaging, cells were transduced with a Cre-dependent *AAV2/2-CAG-FLeX-mitoGFP-t2A-mCherry-WPRE* vector (Charles River, 2.7 × 10^10^ GC/ml final titer in medium). This vector encodes a cytosolic mCherry to label the entire neuron and a tagged GFP to label mitochondria. Mitochondrial targeting of GFP is achieved by fusing the target sequence of *Cox8a* on the N-terminus of GFP. Since this vector is Cre-dependent, to avoid recombination of *Pten* and *Socs3* in WT cells, C57Bl6 cells were used instead of *Pten*^*fl/fl*^*;Socs3*^*fl/fl*^ ones in all experiments depicted in Figs. 4 and 5 for the WT condition.

### In vitro procedures

#### Axonal injury

Axonal injury was performed between 12DIV and 14DIV via vacuum aspiration of the axonal medium. Briefly, a thin gel loading tip was attached to an aquarium pump and used to empty the well of the axonal chamber. Afterward, the tip is moved to the entrance of the axonal channel to remove its medium. The operation is performed twice, once per channel entrance. Immediately after, the axonal chamber is refilled with fresh medium. Vacuum aspiration severed the axons in the axonal channel at ∼100 µm from the exit of the grooves. In all experiments, at 0 and 1 dpi, axons were analyzed in the space between the groove exit and their most distal tip in the axonal channel.

#### Retrograde tracing

Retrograde tracing was performed by supplementing the axonal medium with Alexa-conjugated Cholera Toxin B (CTB-AF555, Cat# C34776; Invitrogen) to a final concentration of 0.1 mg/ml in aCNBA, 48 h before the injury. During CTB exposure, the fluidic grading was increased to 150 μl per well in the somatic chamber and 80 μl per well on the axonal one to prevent the flow of CTB to the axonal side. The CTB-supplemented medium was completely washed and replaced with aCNBA the day after, 24 h before axonal injury.

#### Metabolic pathway inhibition

To determine the relative fractions of ATP production at 1 dpi, we measured the axonal ATP concentration (see below) sequentially in axonal medium (aCNBA), in aCNBA supplemented with 10 µM of oligomycin (Cat# 75351; Merck) and in aCNBA supplemented with 10 µM of oligomycin and 50 mM of 2-deoxyglucose (Cat# D8375; Merck). To locally reduce glycolysis in the axons, right after axotomy we replaced aCNBA with galactose medium (gCNBA: glucose- and pyruvate-free Neurobasal-A [Cat# A2477501; Gibco], 0.27 mM sodium pyruvate [Cat# 11360070; Gibco], 2 mM GlutaMAX, 10 mM HEPES, 2% B27, 5 µM forskolin, 50 µg/ml gentamicin, 20 ng/ml CNTF, 50 ng/ml BDNF). To locally inhibit lactate production within the axons, aCNBA was supplemented with 5 µM of GSK right after axotomy (GSK, Cat# HY-100681; MedchemExpress). During treatment, the small fluidic gradient was reversed to prevent the flow of glucose medium from the somatic chamber to the axonal one.

#### Mitochondrial labeling

Due to spectral incompatibility, for the experiments concerning galactose treatment, when combining the imaging of ATP levels with mitochondrial transport, mitochondria were labeled with MitoTracker Red FM (Cat# M22425; Invitrogen) instead of mitoGFP. On the day of imaging, cells were incubated with 100 nM MitoTracker for 45 min, in medium, in both microfluidic compartments and washed prior to imaging.

### Immunocytochemistry

For immunostaining, cells were fixed for 10 min with 4% paraformaldehyde in PBS (137 mM NaCl, 2.7 mM KCl, 10 mM Na_2_HPO_4_, 1.8 mM KH_2_PO_4_ in distilled water, pH 7.4). All stainings were carried out within the MFDs. Cells were permeabilized with 0.1% Triton-X100 in PBS (PBST), thrice for 5 min. Thereafter, antigen blocking was performed with 10% preimmune donkey serum (PID) in PBST for 45 min or overnight for PFKP, PKM1/2, and LDH. Following, primary antibodies were diluted to the desired concentrations in PBST, supplemented with 10% PID, and incubated overnight at room temperature. For labeling of PTEN, pS6, pSTAT3, PFKP, PKM1/2, and LDHA, the incubation was conducted for 4 days at 4°C with the addition of 10% PID and 0.04% NaN_3_. After primary antibody binding, cells were incubated for 2 h at room temperature with Alexa-conjugated secondary antibodies (1:200 in PBST). Map2 was labeled using an Alexa647 preconjugate primary antibody incubated overnight at 4°C, after the secondary antibodies were bound, to prevent crossreactivity with primary antibodies of the same host. After final washing, the cells were incubated for 30 min with DAPI (1:1,000 in PBS) to label the nuclei. Samples were stored in PBS until imaging, which was performed within 24 h from the staining.

### Primary antibodies

Antibodies were mouse monoclonal anti-tubulin β 3 (TUBB3, clone TUJ1, Cat# 801201, RRID:AB_2313773; BioLegend), mouse monoclonal anti-MAP2 (clone SMI-52, Cat# 801807, RRID:AB_2721423; BioLegend), rabbit polyclonal anti-RBPMS (Cat# 1830-RBPMS, RRID:AB_2492225; PhosphoSolutions), rabbit polyclonal anti-GFAP (Cat# Z0334, RRID:AB_10013382; Agilent), mouse monoclonal anti-GLAST (clone ACSA-1, Cat# 130-095-822, RRID:AB_10829302; Miltenyi Biotec), chicken polyclonal anti-GFP (Cat# ab13970, RRID:AB_300798; Abcam), rabbit monoclonal anti-PTEN (clone 138G6, Cat# 9559, RRID:AB_390810; Cell Signaling Technology), rabbit monoclonal anti-phospho-S6 Ribosomal Protein (Ser235/236) (clone D57.2.2E, Cat# 4858, RRID:AB_2721245; Cell Signaling Technology), rabbit monoclonal anti-phospho-Stat3 (Tyr705) (clone D3A7, Cat# 9145, RRID:AB_2491009; Cell Signaling Technology), rabbit monoclonal anti-PFKP (clone D2E5, Cat# 12746, RRID:AB_2736917; Cell Signaling Technology), rabbit monoclonal anti-PKM1/2 (clone C103A3, Cat# 3190, RRID:AB_2163695; Cell Signaling Technology), rabbit monoclonal anti-LDHA (clone C4B5, Cat# 3582, RRID:AB_2066887, Cell Signaling Technology).

### Imaging

All live imaging was performed on a Zeiss LSM900 with Airyscan 2, with an STXG incubation system (Tokai Hit) maintaining the samples at 5% CO_2_ and 37°C.

#### Epifluorescence imaging

The entire MFD, as in somatic channel, microgrooves, and axonal channel, was imaged before, after injury, and at 1 dpi. Imaging was performed in epifluorescence mode with a Plan-Apochromat 20×/0.8 M27 objective to minimize imaging time and phototoxicity. This way, the whole MFD was imaged in a span of 5 min. Imaging of pS6 and pSTAT3 levels was performed on a Leica DM6 microscope with a HC PL FLUOTAR L 20×/0.40 CORR objective.

#### Confocal imaging

All confocal imaging was performed with a Plan-Apochromat 20×/0.8 M27 objective in CO-2Y mode. Mitochondrial trafficking was imaged via timelapse, carried out for 10 min per field-of-view at a frequency of 0.5 Hz. ATP and lactate biosensor imaging were performed with 405 nm excitation and with emission filters set manually to 450–512 nm for CFP and 512–573 nm for YFP. For both mitochondrial and biosensor imaging, the most distal part of the axon in the axonal channel was imaged. For Tuj1 and MAP2 labeling only, MFD were imaged using an Olympus FV1000 confocal microscope with a UPLSAPO 20×/0.75 objective.

### Image analysis

#### Axonal regeneration

Whole-MFD images of the somatic and axonal compartments at 0 and 1 dpi were aligned via descriptor-based image registration with ImageJ. On these overlaid images, comparing 0 and 1 dpi, axons were counted and scored manually in three classes: (1) regenerating axons, identified by the presence of any measurable amount of extension past the injury site, (2) degenerating axons, which showed complete degeneration or considerable beading and rupture, and (3) static axons, which displayed minimal or no beading, but did not extend past the injury site. After scoring, the length of regenerating axons was measured by manually tracing them with the ImageJ plugin NeuronJ from the most distal tip at 1 dpi until the site of injury (i.e., the distal tip at 0 dpi).

#### Mitochondrial kymographic analysis

Timelapses were stabilized to remove stage drift via descriptor-based image registration with ImageJ. Kymographs were generated by manually tracing the axons from their distal tip through the whole length visible in field-of-view or until the axons were no longer discernible from neighboring ones. Axons that did not present at least ∼25 µm of measurable section from the distal tip were not quantified. Kymographs were analyzed manually using the Kymolyzer set of macros in ImageJ (Basu et al., 2020). Mitochondrial length and occupancy were measured with an ImageJ script. The axon trace from the kymograph analysis was loaded and the axonal mitochondria were segmented via Otsu thresholding. Afterward, the mitochondrial morphological statistics were calculated via the Analyze Particles function. The major axis of the ellipse fit on the segmented particle was used as a measurement of mitochondrial length. Averages of mitochondrial trafficking and length statistics per axon are calculated in Python with the *pandas* library. The mitochondrial moving frequency was defined as the fraction (or percentage) of the entire timelapse duration during which a mitochondrion was classified as motile (Basu et al., 2020). As previously reported (Lewis et al., 2018), mitochondrial axonal occupancy was defined as the fraction of axonal length occupied by mitochondria. Occupancy was calculated as the sum of the length of all mitochondria per axon divided by the axonal length.

#### ATP and lactate analysis

For the analysis of ATP and lactate concentration, the ratios of YFP/CFP and CFP/YFP, respectively, were calculated in Python with the numpy (Harris et al., 2020) and scikit-image (Van Der Walt et al., 2014) libraries after application of a median filter to reduce noise. For quantification, regions of interest were drawn manually within CTB-labeled somata or by tracing the axons from their distal tip through the whole length visible in the field-of-view or until the axons were no longer discernible from neighboring ones. Axons that did not present at least ∼25 µm of measurable section from the distal tip were not quantified. The average ratio intensity per axon or soma was measured in ImageJ. To minimize inter-experiment scattering of the data points, per independent experiment, data points were normalized against the median of their relative naive group. Only for representative images in the figure panels, for ease of visualization and comparison of the pseudocolor, the non-specific FRET signal outside the axons was removed by Otsu thresholding of the YFP signal and using the “Clear Outside” function of ImageJ.

#### Quantification of knock-out efficiency and pathway activation

To quantify the efficiency of AAV-mediated Cre knockout of *Pten* and *Socs3*, the somata of the RGCs were segmented using RGCode, a deep learning tool developed in-house (Masin et al., 2021). For this purpose, a new segmentation model was trained from manual annotations on the dataset. Segmentation was run on images of the entire somatic channel of the MFD. After segmentation, the mean fluorescence intensity of PTEN, pS6, and pSTAT3 was measured per RGC soma. A threshold of mean somatic intensity was manually defined per independent experiment to classify RGCs as marker+ or marker− and calculate their relative percentage.

### Bioinformatic analysis

#### Generation of pseudobulk matrices

Previously published transcriptomic data were obtained from NCBI (GSE202155). The 10× raw data were loaded with scanpy (Wolf et al., 2018) and the *Pten* and *Pten+CNTF* conditions were excluded from the dataset. Prior to pseudobulk generation, quality control was performed to exclude cells that expressed <200 genes and to exclude genes detected in <3 cells. Furthermore, cells with a percentage of mitochondrial genes higher than 5% were excluded, together with cells with >8,000 reads. Only for visualization purposes, cells were projected to Uniform Manifold Approximation and Projection space after principal component analysis. To generate pseudobulk expression matrices, the scRNAseq dataset was split by the biological sample. For conditions having only two biological replicates, each sample was further divided into two pseudoreplicates to aid differential expression analysis. Per replicate, the raw gene reads per cell was summed together to generate a pseudobulk sample. For visualization and quality control, principal component analysis was performed with PyDESeq2 (Muzellec et al., 2023) and plotted with scanpy.

#### Differential expression and GSEA

Differential expression between conditions was performed with PyDESeq2. Genes with low average expression (baseMean < 10) were excluded from the results. Genes were defined as differentially expressed with minimally |log2FC| >1 and FDR <0.05. GSEA was achieved using the *GSEApy* library. Enrichment was performed by querying against the Gene Ontology (GO), Kyoto Encyclopedia of Genes and Genomes (KEGG), and MSigDB databases. The top 10 enriched pathways are represented via dot plots. To visualize gene expression changes upon optic nerve crush (ONC) injury, differentially expressed genes were plotted with hierarchical clustering on heatmaps using the library *seaborn*. To visualize changes in expression related to metabolism specifically, the differentially expressed genes were manually filtered prior to heatmap generation.

### Statistical analysis

Detailed information on the number of samples and axons used is reported in the legends of the figures. The number of *N* axons per condition/time point is reported in the graph under each group. These are accumulated in several independent experiments, of which the specific number is reported in the figure legends. In experiments concerning percentages of axons per microfluidic device, *n* corresponds to the number of independent experiments rather than axons. All data analysis was performed on raw micrographs, which were not saturated during acquisition. For visualization purposes in figure panels, some images were inverted and/or contrast-enhanced by reducing the white point (or increasing the black point when inverted). In this case, the same magnitude of enhancement was applied to all shown images when compared. Statistical tests such as ANOVA, *t* test, and U test were used in combination with bootstrapping for significance testing, and the related details are reported in the figure legends. Estimation statistics with bootstrapping were chosen because of their robustness to large sample sizes, which were required to ensure the accuracy of FRET measurements in axons. The median was used together with Kruskal–Wallis ANOVA if any of the groups in the experiment did not pass the Shapiro–Wilk normality test. Otherwise, the mean and Welch ANOVA were used. As the short recording time leads to zero-inflated data for technical reasons during the measurement of mitochondrial moving frequency, the mean and Welch ANOVA were used despite non-normal data. All numerical data processing, plotting, statistical tests, and bootstrapping were conducted in Python with the libraries pandas, seaborn, matplotlib, scipy, and dabest (Ho et al., 2019). A P value of <0.05 was considered significant.

### Online supplemental material

Fig. S1, related to Fig. 1, shows differentially expressed genes and pathways, recapitulating previously identified genes and validating the analysis. Fig. S2, related to Fig. 3, shows the validation of the microfluidic culture model in terms of cell types present. Moreover, it shows the percentage of degenerating axons after injury. Fig. S3, related to Fig. 3, displays the validation of viral transduction, knockout efficiency, and the related activation of regeneration-associated signaling. Fig. S4, related to Figs. 6, 7, and 8, reports the pairwise comparison of axonal ATP and lactate in uninjured axons between WT and cdKO RGCs, the randomized blind scoring of the axonal labeling of glycolytic enzymes, as well as the relative contribution of mitochondria and glycolysis to the axonal pool of ATP after injury. Fig. S5, related to Figs. 9 and 10, shows the percentage of the different axonal classes after injury during glycolysis inhibition, as well as a comparison in mitochondrial transport, length, and occupancy after injury between cdKO axons in glucose and galactose medium. Moreover, it reports the validation of the inhibition strategy via quantification of axonal lactate. Table S1 contains all the results from the differential expression analysis, as output from PyDESeq2. All comparisons between time points and genotypes are inserted as separate sheets within the file.

## Supplementary Material

Table S1 contains all the results from the differential expression analysis, as output from PyDeSeq2.

## Data Availability

The differential expression data underlying Figs. 1 and 2 are available in the published article and its online supplemental material (Table S1). These were derived from raw sequencing data published by Jacobi et al. (2022) and available in the Gene Expression Omnibus database under accession number GSE202155. Other data are available from the corresponding author upon request. Any additional information required to reanalyze the data is available from the lead contact upon request.
